# Antimicrobial Resistance and Comparative Genome Analysis of High-Risk *Escherichia coli* Strains Isolated from Egyptian Children with Diarrhoea

**DOI:** 10.3390/microorganisms14010247

**Published:** 2026-01-21

**Authors:** Radwa Abdelwahab, Munirah M. Alhammadi, Muhammad Yasir, Ehsan A. Hassan, Entsar H. Ahmed, Nagla H. Abu-Faddan, Enas A. Daef, Stephen J. W. Busby, Douglas F. Browning

**Affiliations:** 1 Institute of Microbiology and Infection, School of Biosciences, University of Birmingham, Birmingham B15 2TT, UK; radwa.wahab418@gmail.com (R.A.); s.j.w.busby@bham.ac.uk (S.J.W.B.); 2 Faculty of Medicine, Assiut University, Assiut 71515, Egypt; dr_ehsan66@aun.edu.eg (E.A.H.); entsar.2012@yahoo.com (E.H.A.); nhi-af@hotmail.com (N.H.A.-F.); deafenas@yahoo.com (E.A.D.); 3 Department of Biology, College of Science, Princess Nourah bint Abdulrahman University, P.O. Box 84428, Riyadh 11671, Saudi Arabia; mmalhammadi@pnu.edu.sa; 4 Quadram Institute Bioscience, Norwich Research Park, Norwich NR4 7UQ, UK; muhammad.yasir@quadram.ac.uk; 5 College of Health and Life Sciences, Aston University, Aston Triangle, Birmingham B4 7ET, UK

**Keywords:** *Escherichia coli*, antibiotic resistance, carbapenemase, virulence, plasmids, whole genome sequencing

## Abstract

*Escherichia coli* is an important human pathogen that is able to cause a variety of infections, which can result in diarrhoea, urinary tract infections, sepsis, and even meningitis, depending on the pathotype of the infecting strain. Like many Gram-negative bacteria, *E. coli* is becoming increasingly resistant to many frontline antibiotics, including third-generation cephalosporins and carbapenems, which are often considered the antibiotics of last resort for these infections. This is particularly the case in Egypt, where multidrug-resistant (MDR) *E. coli* is highly prevalent. However, in spite of this, few Egyptian MDR *E. coli* strains have been fully characterised by genome sequencing. Here, we present the genome sequences of ten highly MDR *E. coli* strains, which were isolated from children who presented with diarrhoea at the Outpatients Clinic of Assiut University Children’s Hospital in Assiut, Egypt. We report that they carry multiple antimicrobial resistance genes, which includes extended spectrum β-lactamase genes, as well as *bla*_NDM_ and *bla*_OXA_ carbapenemase genes, likely encoded on IncX3 and IncF plasmids. Many of these strains were also found to be high-risk extra-intestinal pathogenic *E. coli* (ExPEC) clones belonging to sequence types ST167, ST410, and ST617. Thus, their presence in the Egyptian paediatric population is particularly worrying, and this highlights the need for increased surveillance of high-priority pathogens in this part of the world.

## 1. Introduction

The Gram-negative bacterium *Escherichia coli* is often considered part of the normal gut flora of warm-blooded vertebrate animals, where it acts as a commensal organism [1,2]. However, due to the acquisition of specific virulence genes, some *E. coli* strains can cause disease at various sites within the human body [1,2]. Diarrhoeagenic *E. coli* are important human pathogens, which result in considerable global morbidity and mortality, particularly amongst children in developing countries. These pathogenic strains are grouped into different pathotypes based on their disease characteristics, the toxins they secrete, and their specific adherence patterns, and they include pathotypes such as enteroaggregative *E. coli* (EAEC), enteropathogenic *E. coli* (EPEC), and enterohemorrhagic (EHEC) and enterotoxigenic *E. coli* (ETEC), amongst others [1,2]. Additionally, other *E. coli* strains have acquired specific virulence determinants that enable them to cause extra-intestinal infections, such as urinary tract infections (UTIs), sepsis, and meningitis; these are termed extra-intestinal pathogenic *E. coli* (ExPEC) [1,2]. Thus, pathogenic *E. coli* strains are able to cause a considerable spectrum of disease, depending on their particular genetic makeup.

Like many bacteria, both clinically and environmentally isolated *E. coli* strains are becoming increasingly resistant to many classes of antibiotics, resulting in the emergence of multidrug resistant (MDR) strains. This has led the World Health Organisation (WHO) to categorise antibiotic-resistant Gram-negative bacteria, such as *E. coli*, as high-priority pathogens [3]. In particular, this includes *E. coli* strains that are resistant to third-generation cephalosporin antibiotics (e.g., ceftriaxone) due to the presence of extended-spectrum β-lactamases (ESBLs), as well as strains resistant to carbapenem antibiotics (e.g., imipenem and meropenem), which are often the antibiotics of last resort for many bacterial infections [3,4,5]. Most Carbapenem-Resistant *Enterobacteriaceae* (CRE) carry Ambler class A, B, or D carbapenemases, which include the *Klebsiella pneumoniae* carbapenemase (KPC), the New Delhi metallo-β-lactamase (NDM), and oxacillin-hydrolysing enzymes (OXA-48-like), respectively [4,5]. These resistance genes are often encoded on mobile genetic elements, such as transposons, integrons, and conjugative plasmids, which facilitate their spread, frequently in conjunction with other antimicrobial resistance genes (ARGs). Thus, the global prevalence of CRE is causing a significant public health catastrophe as treatment options dwindle [3,4,5,6,7].

This is particularly the case in Egypt, where CRE are extremely prevalent, being detected at high rates [8,9]. For example, 10.5% of Egyptian isolates from the SMART Global Surveillance Program (2008 to 2014) were positive for carbapenemase carriage [8], whilst 16.1% of all carbapenemase-producing *E. coli* isolated from 36 countries (2015 to 2017) were Egyptian in origin [9]. However, despite this, comparatively few Egyptian carbapenem-resistant *E. coli* strains have been fully characterised by genome sequencing. Of those that have, it is clear that they often carry multiple ESBLs (e.g., *bla*_CTX-M-15_ and *bla*_TEM-1B_) and carbapenemase genes (e.g., *bla*_NDM-1_, *bla*_NDM-5_, *bla*_OXA-48_, and *bla*_OXA-181_), making them resistant to β-lactam antibiotics [9,10,11,12,13,14,15,16]. In many instances, carbapenemase genes are located on conjugative plasmids, co-localised with other ARGs, facilitating their transfer and spread [11,12]. In a previous study, we isolated 50 *E. coli* strains from children (aged from 2 months to 5 years old), who presented with diarrhoea at the Outpatients Clinic of Assiut University Children’s Hospital in 2016 [16]. In this study, we investigated two *E. coli* strains, E36 and E42, which were EAEC in character, using genome sequencing and gene expression analysis to examine virulence determinant regulation [16]. Troublingly, many of the other isolates were resistant to all tested antibiotics, including carbapenems [16]. As patients had not been previously admitted to hospital, this provided us with an opportunity to examine the *E. coli* strains circulating within the paediatric population in Assiut. Thus, to understand more about these strains and to identify the ARGs, plasmids, and virulence determinants they carry, we characterised 10 of these highly MDR isolates using whole genome sequencing and comparative genomic analysis.

## 2. Materials and Methods

### 2.1. Isolation and Characterisation of E. coli Strains

The current work is a retrospective study analysing *E. coli* strains collected in 2016 from infants and children (aged from 2 months to 5 years old) who presented with diarrhoea at the Outpatients Clinic of Assiut University Children’s Hospital [16]. Ethical approval was granted by the Medical School Ethical Review Board before sample collection proceeded [16]. Individuals from this study had frequent watery diarrhoea (>3 times/day), with or without blood or mucus, and participants who had received antibiotics within the last 72 h were excluded from the study. One *E. coli* strain was isolated per patient, and identification was carried out at the Medical Research Center, Faculty of Medicine, Assiut University. Note that due to the stipulations of our ethical approval, no identifiable data was collected to link patients or disease outcomes to any of the samples. In total, ten MDR isolates were chosen for further study, based on the results of the antibiotic susceptibility testing performed in [16].

### 2.2. Genome Sequencing

The draft genome sequencing of each *E. coli* strain was carried out using Illumina sequencing (San Diego, CA, USA) by Microbes NG (https://microbesng.com/ (accessed on 8 November 2025)) as detailed previously [16]. Note that long-read sequencing, such as Oxford Nanopore Technology (Oxford, UK), was not employed due to the limited funding available for this project. Illumina reads were adapter trimmed using Trimmomatic 0.30 with a sliding window quality cutoff of Q15 [17]. Genome assembly was performed using Unicycler v0.4.0 [18] and contigs were annotated using Prokka 1.11 [19]. This Whole Genome Shotgun project has been deposited at DDBJ/ENA/GenBank with the sequence data (BioProject: PRJNA1298299) under the accession numbers: E4: JBQGXB000000000, E15: JBQGXA000000000, E23: JBQGWZ000000000, E27: JBQGWY000000000, E28: JBQGWX000000000, E29: JBQGWW000000000, E30: JBQGWV000000000, E34: JBQGWU000000000, E35: JBQGWT000000000, and E43: JBQGWS000000000.

### 2.3. Bioinformatic Analysis of Genome Sequences

Sequence types were determined using MLST 2.0 [20], bacterial serotyping was determined using SerotypeFinder 2.0 [21], plasmids were identified by detecting plasmid replicons using PlasmidFinder 2.1 [22], and virulence gene analysis was performed using VirulenceFinder 2.0 [23,24] and PathogenFinder 1.1 and 2 [25,26] using software at the Center for Genomic Epidemiology (CGE) (http://www.genomicepidemiology.org/ (accessed on 8 January 2026)). Antibiotic resistance genes were detected using ResFinder version 4.7.2 also at CGE [27] (using ResFinder (22 March 2024) and PointFinder (8 March 2024) databases with settings of 98% and 60% for threshold and length ID, respectively). The phylotype of each strain was determined using the EzClermont in silico Clermont phylotyper version 0.7 (https://ezclermont.hutton.ac.uk/ (accessed on 8 January 2026)) [28]. Insertion sequences and bacteriophage were identified using ISfinder (https://www-is.biotoul.fr/index.php (version 21 November 2025) (accessed on 15 January 2026)) [29] and PHASTER (version 22 December 2020) (https://phaster.ca/ (accessed on 8 November 2025)) [30], respectively.

Draft genomes were visualized with Artemis [31], genomes were compared using the Proksee Server (https://proksee.ca/about (accessed on 8 January 2026)) [32], the Artemis Comparison Tool (ACT) [33], and the Basic Local Alignment Search Tool (BLAST) at NCBI (https://blast.ncbi.nlm.nih.gov/Blast.cgi (accessed on 8 January 2026)). Figures showing genome organization were drawn using ACT [33] and the Proksee Server [32].

**Figure 1:**
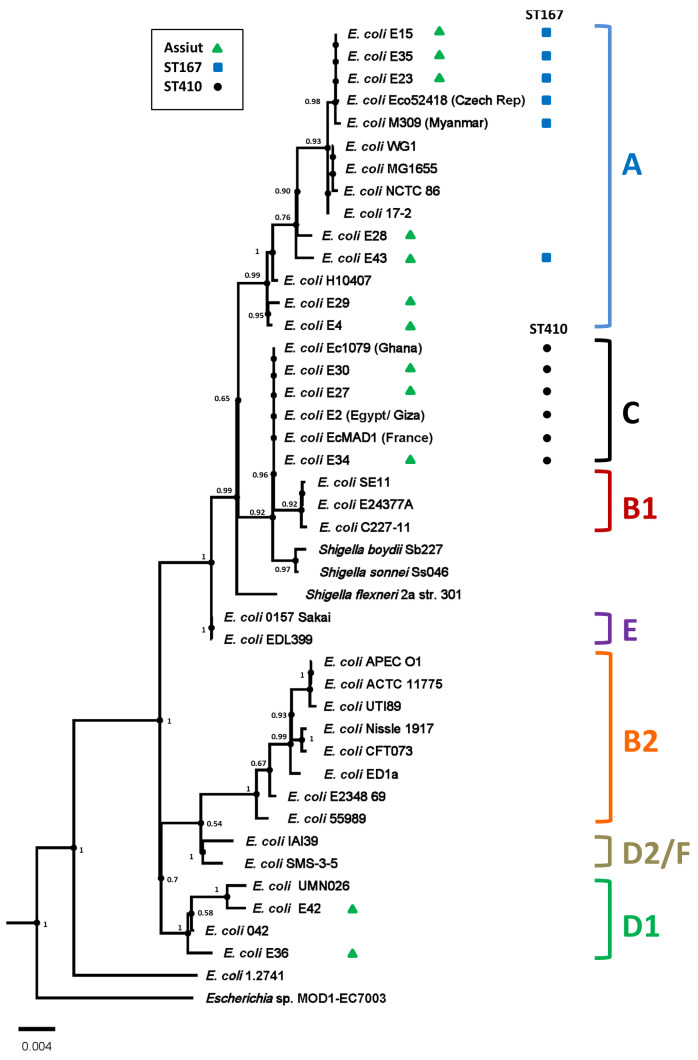
Phylogenetic analysis of the *E. coli* strains isolated in this study. The figure shows a phylogenetic tree of various *E. coli* strains, highlighting the position of the strains investigated in this study (green triangles). The tree was reconstructed from the strains in Abdelwahab et al. [16] using AutoMLST2.0 (https://automlst2.ziemertlab.com/ (accessed on 8 November 2025)) [34]. The various *E. coli* phylotypes are indicated, and sequence types ST167 and ST410 are indicated by blue squares and black dots, respectively. The probability of specific branch points, as defined by AutoMLST2, are also given.

The phylogenetic analysis of strains was carried out by recreating the phylogenetic tree from Abdelwahab et al. [16], using the genomes listed in Figure 1 of that study, and the draft genomes generated in this study. The tree was generated using the AutoMLST2.0 web server for microbial phylogeny (https://automlst2.ziemertlab.com/ (accessed on 8 November 2025)) using the standard Denovo Mode with default settings [34]. The phylogenetic tree was visualized, and the *E. coli* branches selected, using the NCBI Tree Viewer 1.19.0 (https://www.ncbi.nlm.nih.gov/tools/treeviewer/ (accessed on 8 November 2025)). The draft genome sequences of Egyptian *E. coli* strains were obtained by searching the Enterobase Database [35] and the NCBI Pathogen Detection Browser (https://www.ncbi.nlm.nih.gov/pathogens/ (accessed 9 November 2025)) [36]. Single nucleotide polymorphism (SNP) analysis and phylogenetic tree construction was carried out on Egyptian ST167 and ST410 isolates using the pipeline at Solu Genomics (https://www.solugenomics.com/ (accessed on 5 January 2026)) [37]. Briefly, genomes were aligned to the *E. coli* MG1655 reference genome (NC_000913) using Snippy v4.6.0 (https://github.com/tseemann/snippy (accessed on 5 January 2026)). Phylogenetic trees for each species were then inferred with the IQ-TREE v2.3.6 maximum-likelihood algorithm [38]. *E. coli* ST167 and ST410 reference genomes were included in the analysis to validate tree construction [39,40,41].

## 3. Results

### 3.1. Isolation and Genome Characterisation of E. coli Strains

Previously, we isolated 50 *E. coli* strains from children with diarrhoea at the Outpatients Clinic of Assiut University Children’s Hospital [16]. AMR profiling against a range of frontline antimicrobial agents, including carbapenems, indicated that ten of the *E. coli* strains possessed resistance to all agents tested, highlighting the MDR phenotype of these isolates [16,42] (Table 1 and Table S1). To understand more about the plasmids, ARGs, and virulence determinants that each strain possessed, the genomes of these strains were sequenced using short-read Illumina whole genome sequencing (Table 1). As is typical of MDR *E. coli* strains, most strains carried multiple plasmid replicons and, therefore, likely possessed a number of different plasmids (Table 2). Phylogenetic analysis of these MDR strains indicated that four isolates were sequence type ST167, three were ST410, and three were ST46, ST617, and ST361 (Figure 1 and Table 1). It is of note that ST167 and ST410 have now become global high-risk *E. coli* clones, harbouring multiple ARGs [40,43,44,45].

### 3.2. Analysis of Acquired AMR Genes and Chromosomal Point Mutations Associated with AMR

Consistent with their antimicrobial susceptibility pattern (Table 1 and Table S1), each strain carried multiple ARGs (Table 3). All strains possessed genes that would confer resistance to β-lactam antibiotics (e.g., *bla*_CTX-M-15_, *bla*_CMY-2_, *bla*_CMY-42_, *bla*_OXA-1_, *bla*_OXA-9_, and *bla*_TEM-1B_), with the ESBL genes *bla*_CTX-M-15_ and *bla*_TEM-1B_ each found in 8/10 draft genomes (Table 3). Importantly, genes encoding carbapenemases (*bla*_NDM-1_, *bla*_NDM-5_, *bla*_NDM-19_, *bla*_OXA-181_, and *bla*_OXA-244_) were found in six isolates (i.e., E4, E15, E23, E27, E35, and E43), explaining these strains’ resistance to the carbapenem antibiotics imipenem and meropenem (Table 3 and Table S1). Furthermore, genes were detected that would result in resistance to fluoroquinolone (*qnrS1* and *aac*(6′)-Ib-cr), aminoglycoside (*aac*(3)-IId, *aac*(6′)-Ib-cr, *aadA1*, *aadA2*, *aadA5*, *aph*(3′)-Ia, *aph*(3″)-Ib, *aph*(6)-Id and *rmtB*), macrolide (*mphA*), sulphonamide (*sul1* and *sul2*), trimethoprim (*dfrA1*, *dfrA12*, *dfrA14* and *dfrA17*), tetracycline (*tetA* and *tetB*), and chloramphenicol (*catB3*) antibiotics (Table 3) [27,46,47]. In addition, most strains possessed chromosomal point mutation in *gyrA*, *parC*, and *parE* that are associated quinolone resistance (i.e., resistance to nalidixic acid and ciprofloxacin) [27,46,47] (Supplementary Table S2). As 9/10 isolates also possess either *qnrS1* and/or *aac*(6′)-Ib-cr genes, in addition to these chromosomal point mutations, this explains the resistance of all strains to ciprofloxacin [27,46,47]. Similarly, 9/10 strains possessed *tet* resistance genes, explaining the high level of tetracycline resistance observed (Table 1, Table 3 and Table S1). Thus, the identification of these resistance determinants correlates with the MDR phenotype observed for these 10 isolates.

### 3.3. Characterisation of IncX3 Plasmids Carrying Carbapenem Resistance Determinants

**Figure 2:**
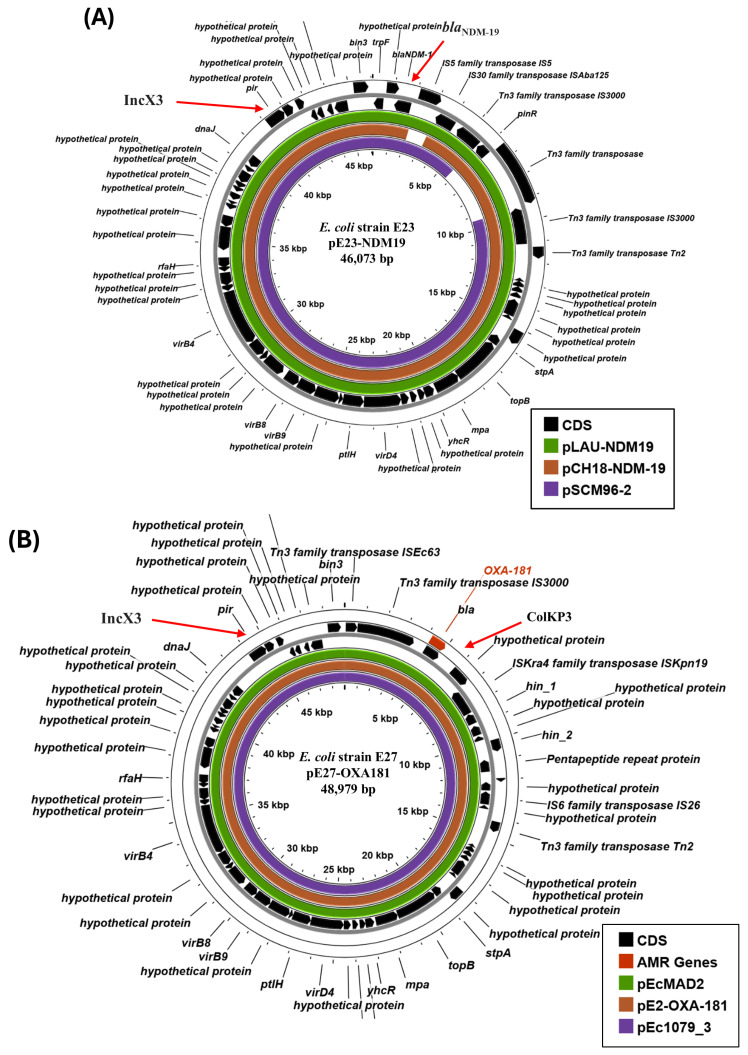
Analysis of the IncX3 plasmids carried by *E. coli* isolates E23 and E27. (**A**) The panel shows the comparison of pE23-NDM19 (E23 contig 27: 46,073 bp) with plasmids pLAU-NDM19 (CP074195.1; 47,332 bp: human isolate) [48], pCH18-NDM-19 (MK091521: 48,737 bp: human isolate) and pSCM96-2 (CP028718.1: 46,161 bp: human isolate) using ProkSee [32]. The genes (CDS) of pE23-NDM19 are displayed in the outer rings, with the location of *bla*_NDM-19_ and the IncX3 replicon indicated by arrows. The green, brown, and purple rings depict the BLAST results when the sequences of pLAU-NDM19, pCH18-NDM-19, and pSCM96-2 are compared with pE23-NDM19. (**B**) The panel shows the comparison of pE27-OXA181 (E27 contig 22: 48,979 bp) with plasmids pEcMAD2 (LR595693.1: 51,479 bp: source unknown) [49], pE2-OXA-181 (CP048918.1: 51,479 bp: human isolate) [10] and pEc1079_3 (CP081309.1: 51,479 bp: human isolate) [50] using ProkSee [32]. The genes (CDS) of pE27-OXA181 are displayed in the outer rings, with the location of *bla*_OXA-181_ and the IncX3 and ColKP3 replicons indicated by arrows. The green, brown, and purple rings depict the BLAST results when the sequences of pEcMAD2, pE2-OXA-181, and pEc1079_3 are compared with pE27-OXA181.

As carbapenem antibiotics are considered the last line of defence against many bacterial pathogens, we sought to understand more about the carbapenemase genes that our strains carry, in particular focusing on the plasmids that might harbour them and lead to their dissemination. Analysis indicated that isolate E23 carries both the *bla*_NDM-19_ carbapenemase gene and the IncX3 plasmid replicon on a single large contig (contig 27: 46,073 bp), suggesting that this might represent a complete plasmid (Table 2 and Table 3). BLAST analysis indicated that this contig was identical (100% coverage: 100% identity) to plasmid pLAU-NDM19 (CP074195.1: 47,332 bp: human isolate), which was isolated in Lebanon from *E. coli* strain EC20 in 2018 [48] (Figure 2A and Figure S1). Plasmid pLAU-NDM19 was shown to be conjugative and strain EC20 was also sequence type ST167. Thus, we propose that strain E23 carries a similar *bla*_NDM-19_-encoding IncX3 plasmid, which we have termed pE23-NDM19 (Figure 2A).

Out of all our strains, E27 carries the most plasmid replicons, possessing seven in total (Table 2). In this instance, the *bla*_OXA-181_ carbapenemase gene co-localises with the IncX3 and ColKP3 replicons on a single large contig (contig 22: 48,979 bp) (Figure 2B). Analysis indicated this contig was identical (100% coverage: 100% identity) to plasmid pE2-OXA-181 (CP048918.1: 51,479 bp: human isolate) isolated in Egypt (Giza) in 2015 [10], plasmid pEcMAD2 (LR595693.1: 51,479 bp: source unknown) isolated in France in 2013 [49], and pEc1079_3 (CP081309.1: 51,479 bp: human isolate) from Ghana in 2015 [50] (Figure 2B and Figure S2). Thus, we propose that strain E27 carries a similar *bla*_OXA-181_-encoding IncX3-ColKP3 plasmid, which we term pE27-OXA181 (Figure 2B). Interestingly, *E. coli* strains E2, EcMAD1, and Ec1079, from which these plasmids came, were sequence type ST410, like E27, and possessed very similar chromosomes with only a few minor regions of difference (Figure 1 and Figure S3 and Table 1) [10,49,50].

### 3.4. Characterisation of IncF Plasmids Potentially Carrying Carbapenem Resistance Genes

**Figure 3:**
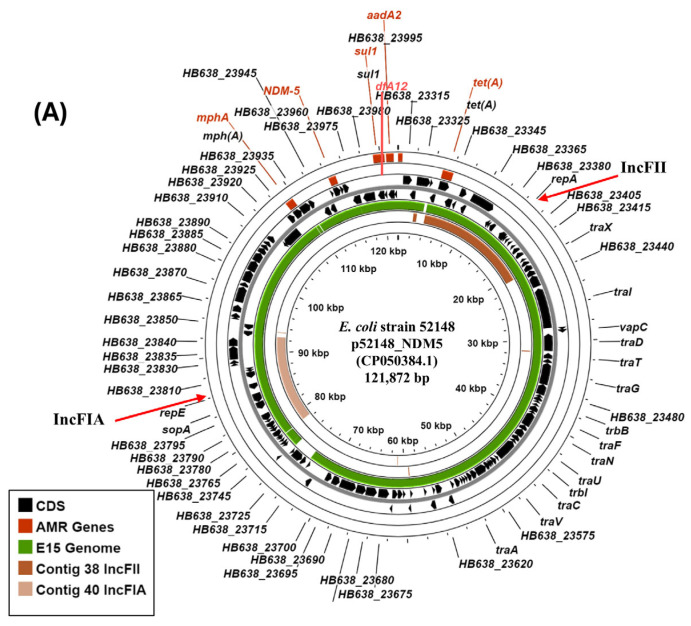

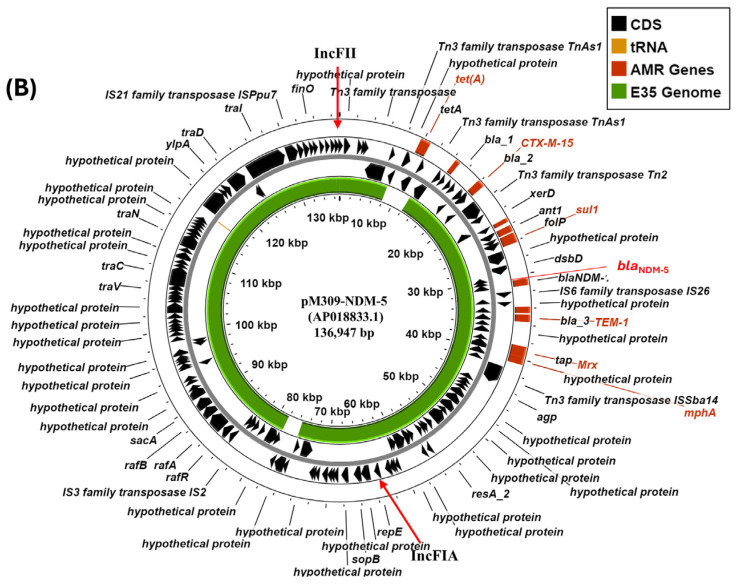
Analysis of IncF plasmids carrying *bla*_NDM-5_ carbapenemase genes in *E. coli* isolates E15 and E35. (**A**) Comparison of *E. coli* Eco52148 plasmid p52148_NDM5 with the draft genome of E15. The panel shows the comparison of p52148_NDM5 (CP050384.1: 121,872 bp: human isolate) [51] with the draft genome of E15 and E15 contigs 38 (16,561 bp) and 40 (14,118 bp), using ProkSee [32]. The outer two rings display the genes of p52148_NDM5 (CDS) on both strands. The green, brown, and light brown rings illustrate the BLAST results when the E15 draft genome and contigs 38 and 40, respectively, are compared to p52148_NDM5. The location of the *bla*_NDM-5_, *mphA*, *sul1*, *aadA2*, *dfrA12*, and *tetA* AMR genes and the IncFII and IncFIA plasmid replicons are shown. (**B**) Comparison of plasmid pM309-NDM5 with the draft genome sequence of *E. coli* isolate E35. The figure shows the comparison of pM309-NDM5 (AP018833.1: 136,947 bp: human isolate) [52] with the draft genome of E35, using ProkSee [32]. The genes (CDS) of pM309-NDM5 are displayed in the outer rings, with the location of the various AMR genes (including *bla*_NDM-5_) and plasmid replicons (IncFIA and IncFII) indicated. The green ring depicts the BLAST results when the E35 draft genome is compared with pM309-NDM5.

Due to the limitations of short-read sequencing, the carbapenemase genes carried by other strains were not co-localised on contigs that possessed plasmid replicons. However, in some instances, we are able to make inferences concerning the plasmids that might harbour these genes. Our analysis indicated that for isolate E15 (sequence type ST167), the *bla*_NDM-5_ gene was located on a small contig (contig 53: 3163 bp), whilst E15 carried three plasmid replicons (Table 2). BLASTn analysis of the IncFII (contig 38: 16,561 bp) and the IncFIA (contig 40: 14,118 bp) replicons indicated that they were very similar to sections of plasmid p52148_NDM5 (CP050384.1: 121,872 bp: human isolate), which also carries *bla*_NDM-5_ (Figure 3A) (100%/99% coverage: 99.99%/99.96% identity, respectively). Strain Eco52148 was isolated in 2019 from a patient who was repatriated from northern Africa to the Czech Republic and, like E15, was sequence type ST167 (Figure 1) [51]. As both p52148_NDM5 and E15 carry *mphA*, *sul1*, *aadA2*, *dfrA12*, and *tetA* ARGs (Table 3 and Figure 3A), we propose that E15 might carry a similar IncFII-IncFIA resistance plasmid.

Strain E35 (also ST167) carries the *bla*_NDM-5_ carbapenemase gene on contig 38 (38,086 bp), which co-localises with *bla*_CTX-M-15_, *bla*_TEM-1B_, *mphA*, *rmtB*, *aadA2*, *sul1*, and *dfrA12* ARGs. BLASTn analysis indicated that this contig was identical (100% coverage: 100% identity) to plasmid pM309-NDM5, carried by ST167 *E. coli* strain M309 (AP018833.1: 136,947 bp), isolated from a patient in Yangon, Myanmar in 2015 (Figure 3B and Figure S4) [52]. Furthermore, E35 contigs carrying the IncFIA (contig 45: 15,794 bp) and IncFII (contig 49: 11,728 bp) replicons were very similar to those carried by pM309-NDM5 (100%/98% coverage: 100%/100%, respectively) (Supplementary Figure S4). Thus, we propose that isolate E35 might carry a large dual IncFIA-IncFII plasmid, which likely encodes *bla*_NDM-5_.

### 3.5. Carriage of Virulence-Associated Genes in Egyptian E. coli Isolates

The analysis of each strain using PathogenFinder indicated that all were potential human pathogens carrying a number of characterised virulence determinants (Supplementary Table S3) [23,24,25,26]. However, none of our strains carried virulence determinants that are specifically associated with diarrhoeagenic *Escherichia coli* pathotypes, such as EAEC, EPEC, EHEC, or ETEC [1,2,53,54]. The virulence factors they did carry included glutamate decarboxylases genes (*gadA*/*gadB*) involved in acid resistance [55]; *iss/bor*, which encodes a lipoprotein involved in increased serum survival [56]; *capU*, a hexosyltransferase homologue [57]; *hra* heat-resistant agglutinin [58]; *traT*, an outer membrane protein involved in serum resistance [59]; and *lpfA* long polar fimbriae [58]. In addition, both E15 and E28 possess the yersiniabactin siderophore uptake system (*ipr2/fyuA*), with E28 carrying additional iron-scavenging systems (i.e., *sitA* iron transport protein and the *iucC*/*iutA* aerobactin system) [60]. It is of note that the carriage of specific virulence genes was sequence type-specific, with ST167 strains (E15, E23, E35, and E43) and ST410 strains (E27, E30, and E34) possessing similar respective virulence profiles (Supplementary Table S3). For ST167 strains, many of the virulence genes they carry (e.g., *iss*, *hra*, *traT*, and *ipr2/fyuA*) are associated with ExPEC [61,62,63] to which sequence type ST167 has been grouped [45]. Interestingly, for E28 (ExPEC sequence type ST617 [40,43,44,45]), *sitABCD* and the aerobactin siderophore gene cluster (*iucABC-iutA*) are located on contig 36 (43,206 bp), flanked by IncFIA and IncFIB replicons, suggesting they are plasmid borne (Supplementary Figure S5). Analysis of this contig and E28 contig 22 (75,303 bp) indicated that they were similar (100%/100% coverage: 100/99.96% identity, respectively) to sections of pEC22-OXA-1 (CP084902.1: 169,208 bp: human isolate) isolated from *E. coli* strain Ec20 in Jinhua, China in 2019. Like pEC22-OXA-1, contig 22 carries *aac(*6′)-Ib-cr, *aadA5*, *bla*_CTX-M-15_, *bla*_OXA-1_, *dfrA17*, two IncFII plasmid replicons, and numerous *tra* genes (Supplementary Figure S5), suggesting that E28 might also carry a hybrid virulence/AMR plasmid potentially capable of conjugative transfer.

### 3.6. SNP Analysis of ST167 and ST410 Egyptian E. coli Isolates

**Figure 4:**
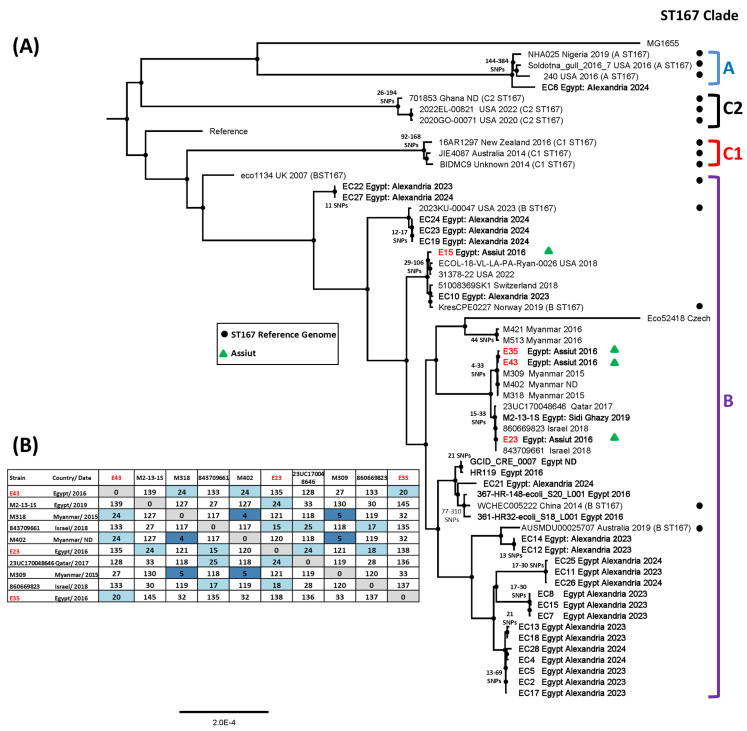
SNP analysis of the *E. coli* ST167 strains isolated in this study. (**A**) The panel shows a phylogenetic tree of various ST167 *E. coli* strains, highlighting the position of the ST167 strains investigated in this study (green triangles). The tree was reconstructed from SNP analysis of the ST167 strains in Supplementary Table S4. The ST167 clades proposed by Walker et al. [39] are indicated, and reference genomes from that study are used to highlight the position of clades A, B, C1, and C2 (indicated by black dots). The names of the 31 Egyptian ST167 are in bold. The range of SNP differences is also given at selected branch points in the tree. ND: no date. (**B**) A SNP distance table showing the pairwise SNP differences for selected strains from (**A**). Light blue shading indicates SNP differences between 25 and 10, and dark blue shading denotes SNP differences below 10.

To understand more about *E. coli* strains isolated in Egypt, we consulted the Enterobase Database, which uses the NCBI Sequence Read Archive, to retrieve the draft genomes of sequenced Egyptian *E. coli* [35]. Using this approach, we identified 125 draft genomes, highlighting the limited genome sequencing of strains within Egypt. By combining this resource with data from published articles and the NCBI Pathogen Detection Browser [36], we were able to identify 32 ST167 and 8 ST410 Egyptian sequenced strains (Supplementary Tables S4 and S5, respectively). Recently, Walker et al. [39] proposed that *E. coli* ST167 could be classified into three major clades (A, B, and C), with clade C resolving into C1 and C2 subclades. SNP analysis, coupled with phylogenetic analysis, indicated that all but one Egyptian ST167 isolate belonged to clade B, clustering with previously identified clade B reference genomes [39] (Figure 4). SNP analysis also indicated that our ST167 isolates, E35 and E43, were closely related, being only separated by 20 SNPs (Figure 4B). Furthermore, isolate E23 was very similar to two Israeli *E. coli* (human isolates 843709661 and 860669823) isolated in Tel Aviv in 2015, differing by only 15 and 18 SNPs, respectively. It is of note that an SNP threshold of ≤25 SNPs has previously been suggested to be indicative of outbreak strains within hospital-based studies [41,64].

**Figure 5:**
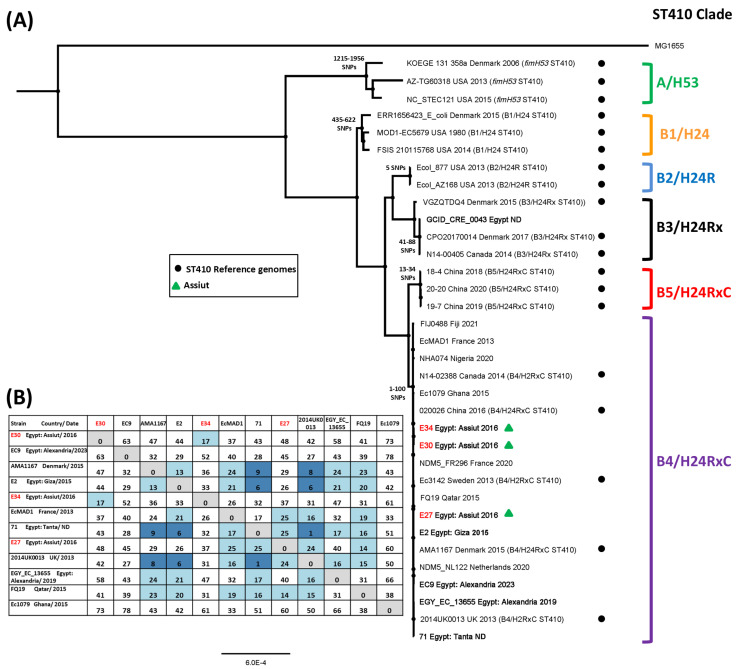
SNP analysis of the *E. coli* ST410 strains isolated in this study. (**A**) The panel shows a phylogenetic tree of various ST410 *E. coli* strains, highlighting the position of the ST410 strains investigated in this study (green triangles). The tree was reconstructed from SNP analysis of the ST410 strains in Supplementary Table S5. The ST410 clades proposed by Roer et al. [40] and Ba et al. [41] are indicated, and reference genomes from these studies are used to highlight the position of clades B1/H24, B2/H24R, B3/H24Rx, B4/H24RxC, and B5/H24RxC) (indicated by black dots). The names of the eight Egyptian ST410 strains are in bold. The range of SNP differences is also given at selected branch points in the tree. ND: no date. (**B**) A SNP distance table showing the pairwise SNP differences for selected strains from (**A**). Light blue shading indicates SNP differences between 25 and 10, and dark blue shading denotes SNP differences below 10.

Sequence analysis of *E. coli* ST410 strains has indicted that the ST410 sequence type comprises two lineages: lineage A, carrying the *fimH53* allele (i.e., A/H53), and lineage B, which possess *fimH24* (B/H24) (Figure 5) [40,41]. Lineage B is divided into five subclades (i.e., clades B1/H24, B2/H24R, B3/H24Rx, B4/H24RxC, and B5/H24RxC), which are based on the sequential appearance of mutations in *parC* and *gyrA*, and the acquisition of various AMR genes (e.g., *bla*_CTX-M-15_ and *bla*_OXA-181_) and plasmids (e.g., IncX3) [40,41]. SNP analysis indicated that all but one Egyptian ST410 isolate belonged to the B4/H24RxC sub-lineage, clustering with previously identified B4/H24RxC reference genomes (Figure 5) [40]. Additionally, analysis indicated that ST410 isolates E30 and E34 were closely related, being separated by only 17 SNPs (Figure 5B). As previously suggested, isolate E27 was found to be very similar to French strain EcMAD1 (25 SNPs: source unknown) and Egyptian strain E2 (Giza: 26 SNPs: human isolate), as well as other human isolates from Egypt (e.g., strain 71 (Tanta): 25 SNPs), the UK (strain 2014UK0013: 24 SNPs), and Quatar (strain FQ19: 14 SNPs). Thus, our analysis supports our suggestion that some of our ST167 and ST410 isolates are very similar to other strains isolated in different countries.

## 4. Discussion

In this study, we characterised ten MDR *E. coli* strains isolated from infants and children with diarrhoea who were treated at the Outpatients Clinic of Assiut University Children’s Hospital in 2016 [16]. It is of note that none of our strains carried virulence determinants that are associated with diarrhoeagenic *Escherichia coli* pathotypes, such as EAEC, EPEC, EHEC, or ETEC [1,53,54]. Rather, they possessed virulence genes associated with the ExPEC pathotype (Supplementary Table S3), with many strains belonging to ExPEC-associated sequence types (i.e., ST167, ST410, and ST617) [40,43,44,45]. As ExPEC strains can colonize and persist in the human gastrointestinal tract without causing disease [65], it is unclear whether the strains we isolated are the cause of diarrhoea in these individuals. However, the human alimentary canal is considered the main reservoir for ExPEC infections [45,65,66]. In the case of UTIs, ExPEC transmission usually follows the faecal-to-perineal/vaginal-to-urethral route, with the possibility of bacteria crossing into the blood stream and causing bacteriemia and sepsis [65,66]. Thus, it is particularly alarming that carbapenem-resistant ExPEC strains were circulating in the paediatric population in Egypt at this time. Importantly, other ExPEC reservoirs include the environment (e.g., water, sewage, etc.), farm animals, meat products, and companion animals, and ExPEC ST167, ST410, and ST617 have all been isolated from such sources (Supplementary Tables S4 and S5) [15,67,68,69,70,71,72,73]. Thus, it is clear that a “One Health Approach”, which takes into consideration human/animal infection and environmental dissemination, is required for control of this pathotype [74].

Previously, we found that many of our original 50 isolates showed some resistance to a number of different classes of antibiotics; for example, 100% of isolates showed resistance to the cephalosporin ceftriaxone, 74% to the fluoroquinolone ciprofloxacin, and 98% to the aminoglycoside tobramycin [16]. In this study, we focused on the strains that possessed extreme MDR phenotypes, demonstrating that each isolate carried numerous ARGs (Table 1, Table 3 and Table S1), and that *bla*_NDM_ or *bla*_OXA_ carbapenemase genes were detected in six isolates. Thus, it is likely that treatment options for infections associated with these strains would be limited, though genome analysis suggests that they remain sensitive to fosfomycin, tigecycline, and colistin. Four of these CRE strains (i.e., E15, E23, E35, and E43) are high-risk ExPEC clones, being sequence type ST167, and all carried variants of the *bla*_NDM_ carbapenemase (Figure 1 and Table 3) [43,44,45]. It is of note that the *bla*_NDM-1_ and *bla*_NDM-5_ alleles have been detected in Egyptian ST167 strains before [9,75]. In spite of the limitations of short-read genome sequencing, we propose that the *bla*_NDM_ genes from strains E15 and E35 may be carried on large IncFIA-IncFII dual replicon plasmids, which is a common occurrence (Figure 3) [4,51,52,75]. Conversely, for strain E23, the *bla*_NDM-19_ carbapenemase gene was encoded on an IncX3 plasmid, pE23-NDM19 (Figure 2A). The NDM-19 carbapenemase was first characterised in 2019, differing from NDM-1 by three amino acid substitutions (i.e., D130N, M154L, and A233V) (Supplementary Figure S1) and conferring high-level resistance to third-generation cephalosporins and carbapenems under zinc-limited conditions, which are thought to prevail at infections sites [76,77]. Thus, its appearance is an important escalation in NDM evolution [77]. Like pE23-NDM19, early isolates carried the *bla*_NDM-19_ gene on conjugational IncX3 plasmids, e.g., pSCM96-2 (CP028718.1: 46,161 bp: human isolate), which was isolated in China in 2017, and pCH18-NDM-19 (MK091521: 48,737 bp: human isolate), which was isolated from an Egyptian patient in Switzerland in 2018 [76,77] (Figure 2A). As our strains predate the isolation of these first plasmids, it is clear that IncX3/*bla*_NDM-19_ plasmids were already present within the Egyptian population as early as 2016.

Three of our strains were sequence type ST410 (i.e., E27, E30, and E34), which is also considered a high-risk ExPEC clone [40,44,45]. Strain E27 carries the *bla*_OXA-181_ carbapenemase on a dual IncX3-ColKP3 plasmid pE27-OXA181, which was similar to plasmids isolated from ST410 strains in France, Egypt, and Ghana (i.e., pEcMAD2, pE2-OXA-181, and pEc1079_3) (Figure 2B and Figure S2) [10,49,50]. Additionally, the chromosomes of these strains (i.e., E2, EcMAD1, and Ec1079) are very similar to those of E27, E30, and E34, with only a few minor regions of difference (Supplementary Figure S3). Our analysis also suggests that these strains likely share additional plasmids. For example, both E27 and E34 carry a p0111 replicon (contigs 18: 92,130 bp and 16: 96,506 bp, respectively) (Table 2), which is also on plasmid pE2-2, carried by strain E2 (Giza/Egypt) (CP048917.1: 92,027 bp) [10] (Supplementary Figure S6). Furthermore, strains EcMAD1, E2, and Ec1079 also carry an IncFIA/IncFIB/IncFII multi-replicon plasmid (i.e., pEcMAD1, pE2-NDM-CTX-M, and pEc1079_1, respectively) that could be detected in the draft genomes of our three ST410 strains (Supplementary Figure S7). Thus, there seems to be a close relationship between these ST140 strains and the plasmids that they carry.

Although we were only able to identify a small number of Egyptian *E. coli* genomes during our database searches due to the limited genome sequencing that has taken place in Egypt, we were able to demonstrate that a considerable proportion of these strains were sequence type ST167 (Supplementary Table S4). SNP analysis indicated that the majority of Egyptian ST167 strains belonged to ST167 clade B (Figure 4), with many isolated in Alexandria between 2023 and 2024. Recently, Walker et al. [39] proposed that ST167 subclade C2 was a highly AMR resistant ST167 clone that was expanding in North America and had also been detected in Europe, Africa, and Asia. The absence of subclade C2 within Egyptian ST167 strains could indicate that this subclade has not established itself within the Egyptian population, or that it has not been detected due to the limited sample size of our study. Irrespective of this, SNP analysis indicated that Assiut strains E35 and E43 were closely related and E23 was very similar to a number of strains including two Israeli human isolates (843709661 and 860669823): strain 23UC170048646 from Qatar (human isolate) and strain M2-13-1, isolated from chicken faeces (Egypt: Sidi Ghazy) (Figure 4B and Table S6) [15]. Interestingly, these strains possess similar virulence and AMR profiles, and like E23, each harbours an IncX3 plasmid, likely carrying *bla*_NDM-19_ (Supplementary Table S6 and Figure S8). Thus, our analysis not only suggests that similar ST167 strains were circulating within Egypt and surrounding countries, but, as demonstrated by Soliman et al. [15], that they had entered the food chain, emphasising the need for a “One Health Approach” to combat AMR in Egypt [74].

With regard to the ST410 strains from our study, we were only able to identify eight Egyptian ST410, suggesting that ST410 is a lesser sequence type within Egypt (Supplementary Table S5). However, in spite of this, our analysis indicates that the majority of Egyptian ST410 strains fall within the ST410 B4/H24RxC sub-lineage and that Assiut strains E30 and E34 were closely related (Figure 5B). Additionally, strain E27 was highly similar to strains from Egypt (E2 (Giza) and 71 (Tanta)), France (EcMAD1), Quatar (FQ19), Denmark (AMA1167), and the UK (2014UK0013) (Figure 5). It is of note that the Danish strain AMA1167 is only separated from the Egyptian strains 71 and E2 by 9 and 14 SNPs, respectively. As AMA1167 was isolated from a patient that had travelled to Egypt, Roer et al. [40] postulated that its acquisition had taken place during that visit, though corroborating sequence data was lacking [78]. Our data lends credence to this hypothesis and their additional suggestion that a similar transition to the UK may have occurred [40,78].

Even though this study was limited by sequencing only a small number of our isolates and would have benefited by combining short-read sequencing with longer-read technologies to generate more complete genome assemblies [79,80], we have still been able to gain a snapshot of the MDR *E. coli* strains present in the paediatric population in Assiut in 2016. It is particularly concerning that, due to the lack of funding and routine sequencing in Egypt [81,82,83], it has taken considerable time for us to uncover that carbapenem-resistant high-risk ExPEC clones were present within the population during this time. Recently, the Egyptian government made considerable inroads into combating AMR with the release of various national guidelines for the rational use of antimicrobials (see [84,85] for examples). However, there is evidence that antibiotics can still be purchased in Egypt without a prescription [86,87,88], and there are cases of unnecessary prescribing, particularly of β-lactams [87,89]. Coupled with this, the increase in AMR resistance associated with humanitarian disasters in nearby places, such as Gaza, will likely continue to place a considerable burden on Egyptian resources [90,91,92]. Thus, it is clear that there is still a need for improved antimicrobial stewardship, infection control, and better surveillance of high-priority Gram-negative CREs to combat their spread in, and from, this geographical area.

## Figures and Tables

**Table 1:** Analysis of the draft genomes and AMR profiles of the *E. coli* strains isolated in this study.

Strain	Genome Size	Number of Contigs	Genes (CDS)	Sequence Type ^a^	Serotype ^b^	Phylotype ^c^	AMR Profile ^d^
**E4**	4,823,577 bp	107	4618	ST46	O8:H4	A	1, 2, 3, 4, 5 *, 6, 7
**E15**	5,004,224 bp	123	4669	ST167	O101:H9	A	1, 2, 3, 4, 5, 6, 7
**E23**	4,937,645 bp	137	4621	ST167	O101:H5	A	1, 2, 3, 4, 5, 6, 7
**E27**	5,007,556 bp	109	4719	ST410	O8:H9	C	1, 2, 3, 4, 5, 6, 7
**E28**	5,025,348 bp	209	4697	ST617	O101:H10	A	1, 2, 3, 4, 5, 6, 7
**E29**	4,959,121 bp	109	4676	ST361	O9:H30	A	1 *, 2, 3, 4, 5, 6, 7
**E30**	4,842,681 bp	105	4531	ST410	O8:H9	C	1 *, 2, 3, 4, 5, 6, 7
**E34**	4,954,276 bp	70	4648	ST410	O8:H9	C	1 *, 2, 3, 4, 5, 6, 7
**E35**	5,039,562 bp	178	4717	ST167	O101:H5	A	1, 2, 3, 4, 5, 6, 7
**E43**	4,921,700 bp	154	4588	ST167	O101:H5	A	1 *, 2, 3, 4, 5, 6, 7

Software at CGE was used to identify: ^a^ the sequence type [20] and ^b^ the serotype [21] of each strain. ^c^ The phylotype of each strain was determined using the EzClermont in silico Clermont phylotyper [28]. ^d^ Key: 1, carbapenem resistance; 2, cephalosporin resistance; 3, penicillin resistance; 4, quinolone resistance; 5, aminoglycoside resistance; 6, tetracycline resistance; 7, trimethoprim sulfonamide resistance. * Intermediate resistance (see Supplementary Table S1). Data on the resistance profile of each strain was previously presented in Abdelwahab et al. [16].

**Table 2:** Analysis of plasmid replicons detected in the draft genomes of the *E. coli* strains isolated in this study.

	Detected Plasmid Replicons ^a^												
Strain	IncFIA	IncFIB	IncFII	IncI	IncQ1	IncY	IncX3	p0111	Col440II	Col(BS512)	ColKP3	Col(MG828)	Number of Replicons
**E4**													**1**
**E15**													**3**
**E23**													**4**
**E27**													**7**
**E28 ^b^**													**6**
**E29**													**4**
**E30**													**4**
**E34**													**5**
**E35**													**4**
**E43**													**3**

^a^ Plasmid replicons were identified using PlasmidFinder software at CGE [22]. Black shading indicates the presence of that replicon. ^b^ Note that strain E28 possesses two IncFII replicons.

**Table 3:** Analysis of AMR genes detected in the draft genomes of *E. coli* strains isolated from children at Assiut Children’s Hospital Outpatients Clinic.

Detected AMR Resistance Genes ^a^																																
	*bla*CTX-M-15	*bla*CMY-2	*bla*CMY-42	*bla*NDM-1	*bla*NDM-5	*bla*NDM-19	*bla*OXA-1	*bla*OXA-9	*bla*OXA-181	*bla*OXA-244	*bla*TEM-1B	*qnrS1*	*aac*(3)-IId	*aac*(6′)-Ib-cr	*aadA1*	*aadA2*	*aadA5*	*aph*(3′)-Ia	*aph*(3″)-Ib	*aph*(6)-Id	*rmtB*	*mph*(A)	*sul1*	*sul2*	*dfrA1*	*dfrA12*	*dfrA14*	*dfrA17*	*tet*(A)	*tet*(B)	*catB3*	Total ARG
**E4**																																**9**
**E15**																																**12**
**E23**																																**8**
**E27**																																**10**
**E28**																																**8**
**E29**																																**9**
**E30**																																**12**
**E34**																																**14**
**E35**																																**11**
**E43**																																**12**

**^a^** The AMR genes carried by each strain were identified using ResFinderFG 4.7.2 software at CGE [27]. Carbapenemase genes are highlighted in red.

## Data Availability

This Whole Genome Shotgun project has been deposited at DDBJ/ENA/GenBank with the sequence data for *E. coli* strains (BioProject: PRJNA1298299) under the accession numbers: E4: JBQGXB000000000, E15: JBQGXA000000000, E23: JBQGWZ000000000, E27: JBQGWY000000000, E28: JBQGWX000000000, E29: JBQGWW000000000, E30: JBQGWV000000000, E34: JBQGWU000000000, E35: JBQGWT000000000 and E43: JBQGWS000000000.

## Supplementary Materials

The following supporting information can be downloaded at: https://www.mdpi.com/article/10.3390/microorganisms14010247/s1, Figure S1: Comparison of *E. coli* plasmid pLAU-NDM19 with the draft genome of *E. coli* E23; Figure S2: Comparison of *E. coli* plasmid pEcMAD2 with the draft genome of *E. coli* E27; Figure S3: Comparison of *E. coli* EcMAD1 and E2 chromosomes with the draft genomes of *E. coli* E27, E30 and E34; Figure S4: Comparison of plasmid pM309-NDM5 with the draft genome of strain E35; Figure S5: Analysis of contigs 22 and 36 from *E. coli* strain E28; Figure S6: Comparison of *E. coli* plasmid pE2-2 with the draft genomes of E. coli E27 and E34; Figure S7: Analysis of plasmid pEcMAD1; Figure S8: Comparison of pE23-NDM19 with the genomes of *E. coli* strains 23UC170048646, 843709661, 860669823 and M2-13-1; Table S1: Antimicrobial susceptibility profile of the MDR *E. coli* strains isolated from children with diarrhoea at Assiut Children’s Hospital, Egypt; Table S2: Analysis of chromosomal point mutations associated with nalidixic acid and ciprofloxacin resistance carried by the *E. coli* strains used in this study; Table S3: Analysis of the potential virulence genes carried by the *E. coli* strains used in this study; Table S4: The *E. coli* ST167 genomes used for phylogenetic SNP analysis in this study; Table S5: *E. coli* ST410 genomes used for phylogenetic SNP analysis in this study; Table S6: Characterisation of *E. coli* ST167 strains related to Egyptian isolate E23. References [10,15,16,20,21,22,23,25,27,28,32,33,35,36,39,40,41,48,49,50,51,52,75,78,93,94,95,96] are cited in the supplementary materials.
