# Supplementary material for: Antimicrobial Resistance and Comparative Genome Analysis of High-Risk Escherichia coli Strains Isolated from Egyptian Children with Diarrhoea
Source: Microorganisms. 2026 Jan 21;14(1):247. doi: 10.3390/microorganisms14010247 (PMC12844379; doi:10.3390/microorganisms14010247)

Edited by DFB on 10/1/2026

Antimicrobial resistance and comparative genome analysis of high-risk *Escherichia coli* strains isolated from Egyptian children with diarrhoea.

Supplementary Material

Radwa Abdelwahab [1, 2], Munirah M. Alhammadi [3], Muhammad Yasir [4],
Ehsan A. Hassan [2], Entsar H. Ahmed [2], Nagla H. Abu-Faddan [2],
Enas A. Daef [2], Stephen J. W. Busby [1] and Douglas F. Browning [5]*

[1] Institute of Microbiology and Infection, School of Biosciences, University of Birmingham, Birmingham, B15 2TT, UK.
[2] Faculty of Medicine, Assiut University, Egypt.
[3] Department of Biology, College of Science, Princess Nourah bint Abdulrahman University, P.O. Box 84428, Riyadh 11671, Saudi Arabia.
[4] Quadram Institute Bioscience, Norwich Research Park, Norwich, NR4 7UQ, UK.
[5] College of Health and Life Sciences, Aston University, Aston Triangle, Birmingham, B4 7ET, UK.

* For correspondence: DFB: Email: d.browning@aston.ac.uk

Table S1. Antimicrobial susceptibility profile of the MDR *E. coli* strains isolated from children with diarrhoea at Assiut Children's Hospital, Egypt.

	Antibiotic [b,c]											
**Strain [a]**	**Ipm**	**Mem**	**Cfc**	**Cro**	**Amc**	**Amp**	**Cip**	**Nor**	**Tob**	**Amk**	**Otc**	**Sxt**
**E4**	+	+	+	+	+	+	+	+/-	+/-	+/-	+	+
**E15**	+	+	+	+	+	+	+	+	+	-	+	+
**E23**	+	+	+	+	+	+	+	+	+	+/-	+	-
**E27**	+	+	+	+	+	+	+	+	+	+/-	+	+
**E28**	+	+	+	+	+	+	+	+	+	+	+	+
**E29**	+/-	+/-	+	+	+	+	+	+	+	+	+	+
**E30**	+/-	+/-	+	+	+	+	+	+	+	+	+	+
**E34**	+	+/-	+	+	+	+	+	+	+	+	+	+
**E35**	+	+	+	+	+	+	+	+	+	+	+	+
**E43**	+/-	+/-	+	+	+/-	+	+	+	+	+	+	+

[a] Antimicrobial susceptibility of these strains was previously reported in Abdelwahab *et al*. [1]
[b] Antibiotics used were as follows: Ipm, imipenem; Mem, meropenem; Cfc, cefaclor; Cro, ceftriaxone; Amc, amoxicillin; Amp, ampicillin; Cip, ciprofloxacin; Nor, norfloxacin; Tob, tobramycin; Amk, amikacin; Otc, oxytetracycline; Sxt, trimethoprim/ sulfamethoxazole.
[c] Antibiotic resistant (+), susceptible (-) and intermediate (+/-), *i.e.* above the point of susceptibility but below the resistant breakpoint [2].

Table S2. Analysis of chromosomal point mutations associated with nalidixic acid and ciprofloxacin resistance carried by the *E. coli* strains used in this study.

Strain	Chromosomal point mutations associated with AMR [a]	ST [b]
**E4**	N/D	ST46
**E15**	*gyrA* S83L D87N , *parC* S80I, *parE* S458A	ST167
**E23**	*gyrA* S83L D87N, *parC* S80I, *parE* S458A	ST167
**E27**	*gyrA* S83L D87N, *parC* S80I, *parE* S458A	ST410
**E28**	*gyrA* S83L D87N, *parC* S80I, *parE* S458A	ST617
**E29**	*gyrA* S83L, D87N, *parC* S80I E84G	ST361
**E30**	*gyrA* S83L D87N, *parC* S80I, *parE* S458A	ST410
**E34**	*gyrA* S83L D87N, *parC* S80I, *parE* S458A	ST410
**E35**	*gyrA* S83L D87N, *parC* S80I, *parE* S458A	ST167
**E43**	*gyrA* S83L D87N, *parC* S80I, *parE* S458A	ST167

Software at the Center for Genomic Epidemiology (CGE) (http://www.genomicepidemiology.org/ (accessed on 8 January 2026)) was used to identify: [a] the chromosomal point mutations associated with nalidixic acid and ciprofloxacin resistance [3] and [b] the sequence type (ST) [4] of each strain. N/D none detected.

Table S3. Analysis of the virulence genes carried by the *E. coli* strains used in this study.

**Strain**	**ST** [a]	**Virulence determinants** [b]	**Pathogen Finder Score** [c]
**E4**	ST46	*gad, iss, terC*	0.929
**E15**	ST167	*capU, gad, hra, iss, terC, traT,* ***fyuA***, ***irp2***	0.925
**E23**	ST167	*capU, gad, hra, iss, terC, traT*	0.928
**E27**	ST410	*gad, lpfA, terC*	0.937
**E28**	ST617	*gad, iss, sitA, terC, traT,* ***fyuA***, ***irp2***, ***iucC***, ***iutA***	0.928
**E29**	ST361	*astA, capU, gad, sitA, terC, traT*	0.93
**E30**	ST410	*gad, lpfA, terC*	0.939
**E34**	ST410	*gad, lpfA, terC*	0.937
**E35**	ST167	*capU, gad, hra, iss, terC, traT*	0.92
**E43**	ST167	*capU, gad, hra, iss, terC, traT*	0.927

Software at the CGE was used to identify: [a] the sequence type (ST) [4] of each strain, [b] the virulence genes they carry [5] and [c] if they were likely a human pathogen using PathogenFinder 1.1 [6]. For PathogenFinder, scores range from 0 to 1 with values closer to 1 indicating that the input organism was predicted as a human pathogen.

Table S4. The *E. coli* ST167 genomes used for phylogenetic SNP analysis in this study.

	Country of Isolation	Strain Name	Accession/ Reference	ST/ clade	Year of Isolation	Notes	Ref
1	USA Boston	240	SRR4294836	A ST167 [b]	2016	Human: ulcer	[7]
2	USA Alaska	Soldotna_gull_2016_7	SRR6376588	A ST167 [b]	2016	Animal: gull faeces	[7]
3	Nigeria	NHA025	SRR13000507	A ST167 [b]	2019	Human	[7]
4	China	WCHEC005222	SRR5153290	B ST167 [b]	2014	Human: rectal swab	[7]
5	UK	eo1703	ERR435237	B ST167 [b]	2007	Human: blood	[7]
6	USA Pennsylvania	31378-22	SRR21351343	B ST167 [b]	2022	Animal: canine faeces	[7]
7	Norway	KresCPE0227	ERR10321715	B ST167 [b]	2019	Human: rectal sample	[7]
8	Australia: Vitoria	AUSMDU00025707	SRR21883691	B ST167 [b]	2019	Human	[7]
9	USA	2023KU-00047	SRR23476193	B ST167 [b]	2023	Human: abscess	[7]
10	Australia: Sydney	JIE4087	SRR6455967	C1 ST167[b]	2014	Human: rectum	[7]
11	ND [a]	BIDMC98	SRR2131466	C1 ST167[b]	2014	Human	[7]
12	New Zealand	16AR1297	SRR10286349	C1 ST167[b]	2016	Human	[7]
13	Ghana	701853	ERR2570974	C2 ST167[b]	ND [a]	Human	[7]
14	USA	2020GO-00071	SRR12903829	C2 ST167[b]	2020	Human	[7]
15	USA	2022EL-00821	SRR22047288	C2 ST167[b]	2022	Human: urine	[7]
16	Qatar: Doha	23UC170048646	SRR12570014	ST167	2017	Human: urine	[8] [d]
17	Israel: Tel Aviv	860669823	ERR9832493	ST167	2018	Human: clinical material	[8] [d]
18	Israel: Tel Aviv	843709661	ERR9832503	ST167	2018	Human: clinical material	[8] [d]
19	Switzerland: Zurich	51008369SK1	CP029973	ST167	2018	Wounded dog	[8] [d]
20	USA: PA	ECOL-18-VL-LA-PA-Ryan-0026	SRR8767398	ST167	2018	Dog	[8] [d]
21	Myanmar: Yangon	M309	DRR140921	ST167	2015	Human: blood	[9]
22	Myanmar: Yangon	M318	DRR140895	ST167	2015	Human: urine	[9]
23	Myanmar: Yangon	M402	DRR111574	ST167	ND	Human: blood	[9]
24	Myanmar: Yangon	M421	DRR111581	ST167	2016	Human: wound	[9]
25	Myanmar: Yangon	M513	DRR111594	ST167	2016	Human: blood	[9]
26	Czech Rep: Ostrava	Eco52148	CP050382	ST167	2019	Human: urine	[10]
27	Egypt	GCID_CRE_0007	SRR8291864	ST167	ND [a]	Human: urine	[11]
28	Egypt	361-HR32-ecoli_S18_L001	Enterobase Assembly ESC_JB5546AA_AS	ST167	2016	Cat	[12]
29	Egypt	367-HR-148-ecoli_S20_L001	Enterobase Assembly ESC_JB5551AA_AS	ST167	2016	Human	[12]
30	Egypt: Alexandria	EC2	SRR33666351	ST167	2023	Human: wound/ pus	[12]
31	Egypt: Alexandria	EC4	SRR33679778	ST167	2024	Human: urine	[12]
32	Egypt: Alexandria	EC5	SRR33679777	ST167	2023	Human: urine	[12]
33	Egypt: Alexandria	EC6	SRR33679774	ST167	2024	Human: wound/ pus	[12]
34	Egypt: Alexandria	EC7	SRR33679773	ST167	2023	Human: blood	[12]
35	Egypt: Alexandria	EC8	SRR33679772	ST167	2023	Human: wound/ pus	[12]
36	Egypt: Alexandria	EC10	SRR33679770	ST167	2023	Human: respiratory tract	[12]
37	Egypt: Alexandria	EC11	SRR33679779	ST167	2023	Human: urine	[12]
38	Egypt: Alexandria	EC12	SRR33679769	ST167	2023	Human: wound/pus	[12]
39	Egypt: Alexandria	EC13	SRR33679768	ST167	2023	Human: sputum	[12]
40	Egypt: Alexandria	EC14	SRR33679776	ST167	2023	Human: urine	[12]
41	Egypt: Alexandria	EC15	SRR33679775	ST167	2023	Human: urine	[12]
42	Egypt: Alexandria	EC17	SRR33690896	ST167	2023	Human: blood	[12]
43	Egypt: Alexandria	EC18	SRR33690892	ST167	2023	Human: blood	[12]
44	Egypt: Alexandria	EC19	SRR33690891	ST167	2024	Human: respiratory tract	[12]
45	Egypt: Alexandria	EC21	SRR33690889	ST167	2023	Human: wound/ pus	[12]
46	Egypt: Alexandria	EC22	SRR33690888	ST167	2023	Human: blood	[12]
47	Egypt: Alexandria	EC23	SRR33690887	ST167	2024	Human: respiratory tract	[12]
48	Egypt: Alexandria	EC24	SRR33690886	ST167	2024	Human: respiratory tract	[12]
49	Egypt: Alexandria	EC25	SRR33690885	ST167	2024	Human: wound/pus	[12]
50	Egypt: Alexandria	EC26	SRR33690895	ST167	2024	Human: urine	[12]
51	Egypt: Alexandria	EC27	SRR33690894	ST167	2024	Human: wound/ pus	[12]
52	Egypt: Alexandria	EC28	SRR33690893	ST167	2024	Human: wound/ pus	[12]
53	Egypt: Assiut	E15	SRR34956587	ST167	2016	Human: faeces	TW [c]
54	Egypt: Assiut	E23	SRR34956586	ST167	2016	Human: faeces	TW [c]
55	Egypt: Assiut	E35	SRR34956580	ST167	2016	Human: faeces	TW [c]
56	Egypt: Assiut	E43	JBQGWS000000000	ST167	2016	Human: faeces	TW [c]
57	Egypt: Mansoura	HR119	QXNX01	ST167	2016	Human	[11]
58	Egypt: Sidi Ghazy	M2-13-1	SRR23972352	ST167	2019	Chicken: faeces	[13]

[a] ND, not determined. [b] ST167 reference strain. [c] TW, this work. [d] NCBI Pathogen detection https://www.ncbi.nlm.nih.gov/pathogens/

Table S5. *E. coli* ST410 genomes used for phylogenetic SNP analysis in this study.

	Country of Isolation	Strain Name	Accession/ Reference	ST/ Clade	Year of Isolation	Notes	Ref
1	USA	NC_STEC121	SRR5470036	ST410 *fimH23* [a]	2015	Poultry	[14]
2	USA	AZ-TG60318	SRR1178257	ST410 *fimH23* [a]	2013	Poultry	[14]
3	Denmark	KOEGE 131 (358a)	SRR785630	ST410 *fimH23* [a]	2006	Human: urine	[14]
4	Denmark	E. coli_R38	ERR1656423	ST410 B1/H24 [a]	2015	Swine	[14]
5	USA	FSIS 210115768	SRR2970227	ST410 B1/H24 [a]	2014	Poultry	[14]
6	USA	MOD1-EC5679	SRR3987983	ST410 B1/H24 [a]	1980	Poultry	[14]
7	USA	Ecol_AZ168	SRR3999080	ST410 B2/H24R [a]	2013	Human	[14]
8	USA	Ecol_877	SRR3999074	ST410 B2/H24R [a]	2013	Human	[14]
9	Denmark	CPO20170014	ERR2652836	ST410 B3/H24Rx [a]	2017	Human	[14]
10	Denmark	VGZQTDQ4	ERR1971770	ST410 B3/H24Rx [a]	2015	Human	[14]
11	Canada	N14-00405	SRR5714040	ST410 B3/H24Rx [a]	2014	Human	[14]
12	Sweden	Ec3142	ERR2652843	ST410 B4/H24RxC[a]	2013	Human	[14]
13	UK	2014UK0013	ERR2652794	ST410 B4/H24RxC [a]	2014	Human	[14]
14	Canada	N14-02388	SRR5714073	ST410 B4/H24RxC [a]	2014	Human	[14]
15	Denmark	AMA1167	CP024801 ERR2652821	ST410 B4/H24RxC [a]	2015	Human: liver (Egyptian travel)	[14, 15]
16	China	020026	SRR6942787	ST410 B4H24RxC [a]	2016	Human	[16]
17	China	18-4	CP123013.1	ST410 B5H24RxC [a]	2018	Human: blood	[17]
18	China	19-7	CP123017.1	ST410 B5H24RxC [a]	2019	Human: blood	[17]
19	China	20-20	CP123029.1	ST410 B5H24RxC [a]	2020	Human: respiratory	[17]
20	Egypt	GCID_CRE_0043	SRR8291886	ST410	ND [b]	Human Urine	[12]
21	Egypt: Alexandria	EC9	SRR33679771	ST410	2023	Human: urine	[12]
22	Egypt: Alexandria	EGY_EC_13655	SRR19440483	ST410	2019	Human: urine	[12]
23	Egypt Assiut	E27	JBQGWY000000000	ST410	2016	Human: faeces	TW [c]
24	Egypt Assiut	E30	JBQGWV000000000	ST410	2016	Human: faeces	TW [c]
25	Egypt Assiut	E34	JBQGWU000000000	ST410	2016	Human: faeces	TW [c]
26	Egypt Giza	E2	CP048915.1	ST410	2015	Human: ascitic fluid	[18]
27	Egypt Tanta	71	SRR8885623	ST410	ND [b]	Human	[8] [d]
28	France	EcMAD1	LR595691.1	ST410	2013	Source unknown	[19]
29	France	NDM5_FR296	ERR11260640	ST410	2020	Human	[20]
30	Fiji	FIJ0488	SRR31395890	ST410	2021	Human: urine	[21]
31	Ghana	Ec1079	CP081306.1	ST410	2015	Human: urine	[22]
32	Netherlands	NDM5_NL122	ERR11256510	ST410	2020	Human	[20]
33	Nigeria	NHA074	SRR13001296	ST410	2020	Human	[8] [d]
34	Qatar	FQ19	SRR12569983	ST410	2015	Human: urine	[8] [d]

[a] ST410 reference strains. [b] ND, not determined. [c] TW, this work. [d] NCBI Pathogen detection https://www.ncbi.nlm.nih.gov/pathogens/

Table S6. Characterisation of *E. coli* ST167 strains related to Egyptian isolate E23.

**Strain**	**E23**	**23UC170048646**	**843709661**	**860669823**	**M2-13-1**
**Location**	Egypt: Assiut	Qatar: Doha	Israel: Tel Aviv	Israel: Tel Aviv	Egypt: Sidi Ghazy
**Date**	2016	2017	2018	2018	2019
**Source**	Human: faeces	Human: urine	Human: clinical material	Human: clinical material	Chicken: faeces
**Phylotype [a]**	A	A	A	A	A
**Sequence Type [b]**	ST167	ST167	ST167	ST167	ST167
**Serotype [c]**	O101:H5	O101:H5	O101:H5	O101:H5	O101:H5
**Pathogen Finder Score [d]**	**0.928**	**0.929**	**0.930**	**0.930**	**0.922**
**Plasmid replicons [e]**	**IncX3**, IncY Col440II, Col(BS512	**IncX3**, IncY Col440II, Col(BS512	**IncX3**, IncY Col440II, Col(BS512	**IncX3**, IncY Col440II, Col(BS512	**IncX3**, IncY Col440II, Col(BS512 IncFII, IncFIB, IncI1-I
**Antibiotic resistance genes[f]**	*aph*(3")-Ib, *aph*(6)-Id, ***bla***$_{\mathbf{NDM\text{-}19}}$, *bla*$_{\text{CTX-M-15}}$, *bla*$_{\text{TEM-1B}}$, *qnrS1, sul2, tetA*	*aph*(3")-Ib, *aph*(6)-Id, ***bla***$_{\mathbf{NDM\text{-}19}}$, *bla*$_{\text{CTX-M-15}}$, *bla*$_{\text{TEM-1B}}$, *qnrS1, sul2, tetA*	*aph*(3")-Ib, *aph*(6)-Id, ***bla***$_{\mathbf{NDM\text{-}19}}$, *bla*$_{\text{CTX-M-15}}$, *bla*$_{\text{TEM-1B}}$, *qnrS1, sul2, tetA*	*aph*(3")-Ib, *aph*(6)-Id, ***bla***$_{\mathbf{NDM\text{-}19}}$, *bla*$_{\text{CTX-M-15}}$, *bla*$_{\text{TEM-1B}}$, *qnrS1, sul2, tetA*	*aac*(3)-IV, *aph*(3")-Ib, *aph*(3')-Ia *aph*(4)-Ia *aph*(6)-Id, ***bla***$_{\mathbf{NDM\text{-}19}}$, *bla*$_{\text{TEM-1B}}$, *dfrA14, floR, qnrS11, sul2, tetA*
**Chromosomal point mutations associated with AMR[g]**	*gyrA* S83L D87N, *parC* S80I, *parE* S458A	*gyrA* S83L D87N, *parC* S80I, *parE* S458A	*gyrA* S83L D87N, *parC* S80I, *parE* S458A	*gyrA* S83L D87N, *parC* S80I, *parE* S458A	*gyrA* S83L D87N, *parC* S80I, *parE* S458A
**Virulence genes[g]**	*capU, gad, hra, iss, terC, traT*	*capU, gad, hra, iss, terC*	*capU, gad, hra, iss, terC*	*capU, gad, hra, iss, terC*	*capU, gad, hra, iss, terC, traT, traJ*

[a] The phylotype of each strain was determined using the EzClermont *in silico* Clermont phylotyper [23]. Software at CGE was used to identify: [b] the sequence type of each strain [4], [c] their serotype [24], [d] whether strains were likely human pathogens [6], [e] the plasmid replicons they carry [25], [f] the AMR genes and chromosomal point mutations associated with AMR they possess [3] and [g] the virulence determinants each strains carries [5].

Supplementary Figure legends

Figure S1. Comparison of *E. coli* plasmid pLAU-NDM19 with the draft genome of *E. coli* E23. **A)** The panel shows the comparison of pLAU-NDM19 (CP074195.1: human isolate) [26] with the draft genome of E23 and E23 contig 27 (46,073 bp) using ProkSee [27]. The outer two rings display the genes of pLAU-NDM19 (CDS) on both strands. The green and brown rings illustrate the BLAST results when the E23 draft genome and contig 27, respectively, are compared to pLAU-NDM19. **B)** The panel shows the alignment of pLAU-NDM19 (CP074195.1) [26] with E23 contig 27 (46,073 bp) using ACT [28]. Alignment is shown by blue banding, which indicates that the sequences are inverted with respect to each other. The location of the $bla_{\text{NDM-19}}$ in both panels is indicated. **C)** The panel shows the alignment of the amino acid sequences of the NDM-1 (AHM26723), NDM-5 (JN104597), NDM-7 (AKN35289) and NDM-19 (WP_094009810.1) carbapenemases with the NDM-19 carbapenemase carried by *E. coli* strain E23. Differences from NDM-1 are highlighted red.

Figure S2. Comparison of *E. coli* plasmid pEcMAD2 with the draft genome of *E. coli* E27. **A)** The panel shows the comparison of pEcMAD2 (LR595693.1: strain isolation source unknown) [19] with the draft genome of E27, E27 contig 22 (48,979 bp), plasmid pE2-OXA-181 (CP048918.1: human isolate) [18] and plasmid pEc1079_3 (CP081309.1: human isolate) [22],using ProkSee [27]. The outer rings display the genes of pEcMAD2 (CDS) on both strands. The green, light green, brown and blue rings illustrate the BLAST results when the E27 draft genome, E27 contig 22, pE2-OXA-181 and pEc1079_3 are compared to pEcMAD2. **B)** The panel shows the alignment of pEcMAD2 (LR595693.1) [19] with E27 contig 22 (48,979 bp) using ACT [28]. Alignment is shown by red banding. The location of the $bla_{\text{OXA-181}}$ and the IncX3 and ColKP3 replicons panels is indicated in both.

Figure S3. Comparison of *E. coli* EcMAD1 and E2 chromosomes with the draft genomes of *E. coli* E27, E30 and E34. **A)** The panel shows the comparison of the EcMAD1 chromosome (LR595691.1: 4,747,851 bp: strain source unknown) [19] with the draft genomes of E27, E30 and E34, and the chromosomes of *E. coli* strains E2 (CP048915.1: human isolate) [18] and Ec1079 (CP081306.1: human isolate) [22], using ProkSee [27]. The outer rings display the genes of EcMAD1 (CDS) on both strands. The green, brown, purple, orange and blue rings illustrate the BLAST results when the E27, E30 and E34 draft genomes and the chromosomes of *E. coli* strains E2 and Ec1079 are compared to EcMAD1. **B)** The panel shows the comparison of the E2 chromosome (CP048915.1: 4,741,120 bp) [18] with the draft genomes of E27, E30, E34, and the chromosomes of *E. coli* strains EcMAD1 (LR595691.1 bp) [19] and Ec1079 (CP081306.1) [22], using ProkSee [27]. The outer rings display the genes of E2 (CDS) on both strands. The green, brown,

purple, orange and blue rings illustrate the BLAST results when the E27, E30 and E34 draft genomes, and the chromosomes of *E. coli* strains EcMAD1 and Ec1079 are compared to E2.

Figure S4. Comparison of plasmid pM309-NDM5 with the draft genome of stain E35. **A)** The panel shows the comparison of pM309-NDM5 (AP018833.1: 136,947 bp: human isolate) [9] with the draft genome of E35 and E35 contigs 38 (38,086 bp), 45 (IncFIA replicon: 15,794 bp) and 49 (IncFII replicon: 11,728 bp), using ProkSee [27]. The genes (CDS) of pM309-NDM5 are displayed in the outer rings, with the location of the various AMR genes (including $bla_{\text{NDM-5}}$) and plasmid replicons (IncFIA and IncFII) indicated. The green, brown, light brown and light green rings depict the BLAST results when the E35 draft genome and contigs 38, 45 and 49 are compared with pM309-NDM5. **B)** The panel shows the alignment of pM309-NDM5 (AP018833.1) [9] with E35 contig 38 (38,086 bp) using ACT [28]. Alignment is shown by blue banding, which indicates that the sequences are inverted with respect to each other. The location of the $bla_{\text{NDM-5}}$ is indicated.

Figure S5. Analysis of contigs 22 and 36 from *E. coli* strain E28. Genomic organisation of E28 **A)** contig 36 (43,206 bp) and **B)** contig 22 (75,303 bp) using ProkSee [27]. **C)** Comparison of plasmid pEC22-OXA-1 from *E. coli* strain Ec20 with the draft genome of *E. coli* E28. The panel shows the comparison of pEC22-OXA-1 (CP084902.1: 169208 bp: human isolate) with the draft genome of E28, E28 contigs 22 (75,303 bp) and 36 (43,206 bp), and plasmid pEC22-CTX-M-15 (CP157417.1: 169,208 bp: human isolate), using ProkSee [27]. The outer two rings display the genes of pEC22-OXA-1 (CDS) on both strands. The green, brown, light brown and purple rings illustrate the BLAST results when the E28 draft genome, contigs 22 and 36, and pEC22-CTX-M-15, respectively, are compared to pEC22-OXA-1. **D)** The panel shows the alignment of pEC22-OXA-1 (CP084902.1: 169,208 bp) with E28 contigs 22 (75,303 bp) and 36 (43,206 bp) using ACT [28]. Alignment is shown by red and blue banding.

Figure S6. Comparison of *E. coli* plasmid pE2-2 with the draft genomes of *E. coli* E27 and E34. **A)** The panel shows the comparison of plasmid pE2-2 (CP048917.1: 92,027 bp: human isolate) [18] with the with the draft genome and contig 28 (92,136 bp) of E27, and the draft genome of E34 and contig 16 (96,506 bp), using ProkSee [27]. The outer rings display the CDS of pE2-2 on both strands. The green, light green, brown and light brown rings illustrate the BLAST results when the E27 draft genome, E27 contig 18, E34 draft genome and E34 contig 16, respectively, are compared to pE2-2. **B)** The panel shows the alignment of E34 contig 16 (96,506 bp), E27 contig 18 (92,136 bp) with pE2-2 (CP048917.1) [18], using ACT [28]. Alignment is shown by red and blue banding. Blue banding indicates that the sequences have been inverted with respect to each other. The location of the p0111 replicon is indicated in both panels.

Figure S7. Analysis of *E. coli* plasmid pEcMAD1. The panel shows the comparison of plasmid pEcMAD1 (LR595692.1: 98,473 bp: strain source unknown) [19] with the with the draft genomes of E27, E30 and E34 and plasmids pE2-NDM-CTX-M (CP048916.1: human isolate) [18] and pEc1079_1 (CP081307.1: human isolate) [22], using ProkSee [27]. The outer rings display the CDS of pEcMAD1 on both strands. The green, brown, purple, orange and blue rings illustrate the BLAST results when pEcMAD1 is compared to the draft genomes of E27, E30 and E34 and pE2-NDM-CTX-M and pEc1079_1.

Figure S8. Comparison of pE23-NDM19 with the genomes of *E. coli* strains 23UC170048646, 843709661, 860669823 and M2-13-1. (A) The panel shows the comparison of pE23-NDM19 (E23 contig 27: 46,073 bp) with the genomes of *E. coli* strains 23UC170048646 (SRR12570014: Qatar: human isolate), 843709661 (ERR9832503:Israel: human isolate), 860669823 (ERR9832493: Israel: human isolate), and M2-13-1 (SRR23972352: Egypt: chicken isolate) as well as 23UC170048646 contig 32 (47072 bp) and M2-13-1 contig 34 (47300 bp) using ProkSee [27]. **B)** The panel shows the alignment of pE23-NDM19 (E23 contig 27: 46,073 bp) with 23UC170048646 contig 32 (47072 bp) and M2-13-1 contig 34 (47300 bp) using ACT [28]. Alignment is shown by red banding. The location of the $\textit{bla}_{\text{NDM-19}}$ and the IncX3 replicon is indicated in both panels.

Supplementary References

[1] R. Abdelwahab, M. Yasir, R.E. Godfrey, G.S. Christie, S.J. Element, F. Saville, et al., Antimicrobial resistance and gene regulation in Enteroaggregative *Escherichia coli f*rom Egyptian children with diarrhoea: Similarities and differences, Virulence. 12 (2021) 57-74. doi: 10.1080/21505594.2020.1859852.
[2] Clinical_and_Laboratory_Standards_Institute_(CLSI), Performance Standards for Antimicrobial Susceptibility Testing; Twenty-Fourth Informational Supplement, CLSI Document M100-S24, Wayne. 34(1) (2014).
[3] V. Bortolaia, R.S. Kaas, E. Ruppe, M.C. Roberts, S. Schwarz, V. Cattoir, et al., ResFinder 4.0 for predictions of phenotypes from genotypes, J Antimicrob Chemother. 75 (2020) 3491-500. doi: 10.1093/jac/dkaa345.
[4] M.V. Larsen, S. Cosentino, S. Rasmussen, C. Friis, H. Hasman, R.L. Marvig, et al., Multilocus sequence typing of total-genome-sequenced bacteria, J Clin Microbiol. 50 (2012) 1355-61. doi: 10.1128/jcm.06094-11.
[5] K.G. Joensen, F. Scheutz, O. Lund, H. Hasman, R.S. Kaas, E.M. Nielsen, et al., Real-time whole-genome sequencing for routine typing, surveillance, and outbreak detection of verotoxigenic *Escherichia coli*, J Clin Microbiol. 52 (2014) 1501-10. doi: 10.1128/jcm.03617-13.
[6] S. Cosentino, M. Voldby Larsen, F. Møller Aarestrup, O. Lund, PathogenFinder--distinguishing friend from foe using bacterial whole genome sequence data, PLoS One. 8 (2013) e77302. doi: 10.1371/journal.pone.0077302.
[7] L.L. Walker, M.D. Phan, B. Permana, Z.J. Lian, N.T.K. Nhu, T. Cuddihy, et al., Emergence of a carbapenem-resistant atypical uropathogenic Escherichia coli clone as an increasing cause of urinary tract infection, Nat Commun. 16 (2025) 8200. doi: 10.1038/s41467-025-63477-0.
[8] The_NCBI_Pathogen_Detection_Project. National Center for Biotechnology Information, National Library of Medicine, National Institutes of Health, Bethesda, MD 20894, USA. 2016. (Accessed on 9 November 2025). https://www.ncbi.nlm.nih.gov/pathogens/ ed.
[9] Y. Sugawara, Y. Akeda, H. Hagiya, N. Sakamoto, D. Takeuchi, R.K. Shanmugakani, et al., Spreading Patterns of NDM-Producing Enterobacteriaceae in Clinical and Environmental Settings in Yangon, Myanmar, Antimicrob Agents Chemother. 63 (2019). doi: 10.1128/aac.01924-18.
[10] K. Chudejova, L. Kraftova, V. Mattioni Marchetti, J. Hrabak, C.C. Papagiannitsis, I. Bitar, Genetic Plurality of OXA/NDM-Encoding Features Characterized From Enterobacterales Recovered From Czech Hospitals, Front Microbiol. 12 (2021) 641415. doi: 10.3389/fmicb.2021.641415.
[11] L. Li, Y. Zhang, H. Guo, J. Yang, F. He, Genomic insights into a *bla*(NDM-5)-carrying *Escherichia coli* ST167 isolate recovered from faecal sample of a healthy individual in China, J Glob Antimicrob Resist. 36 (2024) 240-3. doi: 10.1016/j.jgar.2023.12.032.
[12] N.P. Dyer, B. Päuker, L. Baxter, A. Gupta, B. Bunk, J. Overmann, et al., EnteroBase in 2025: exploring the genomic epidemiology of bacterial pathogens, Nucleic Acids Res. 53 (2025) D757-d62. doi: 10.1093/nar/gkae902.
[13] A.M. Soliman, H. Ramadan, T. Shimamoto, T. Komatsu, F. Maruyama, T. Shimamoto, Detection and Genomic Characteristics of NDM-19- and QnrS11-Producing O101:H5 *Escherichia coli* Strain Phylogroup A: ST167 from a Poultry Farm in Egypt, Microorganisms. 13 (2025). doi: 10.3390/microorganisms13081769.
[14] L. Roer, S. Overballe-Petersen, F. Hansen, K. Schønning, M. Wang, B.L. Røder, et al.,

Escherichia coli Sequence Type 410 Is Causing New International High-Risk Clones, mSphere. 3 (2018). doi: 10.1128/mSphere.00337-18.
[15] S. Overballe-Petersen, L. Roer, K. Ng, F. Hansen, U.S. Justesen, L.P. Andersen, et al., Complete Nucleotide Sequence of an *Escherichia coli* Sequence Type 410 Strain Carrying bla(NDM-5) on an IncF Multidrug Resistance Plasmid and bla(OXA-181) on an IncX3 Plasmid, Genome Announc. 6 (2018). doi: 10.1128/genomeA.01542-17.
[16] Y. Feng, L. Liu, J. Lin, K. Ma, H. Long, L. Wei, et al., Key evolutionary events in the emergence of a globally disseminated, carbapenem resistant clone in the *Escherichia coli* ST410 lineage, Commun Biol. 2 (2019) 322. doi: 10.1038/s42003-019-0569-1.
[17] X. Ba, Y. Guo, R.A. Moran, E.L. Doughty, B. Liu, L. Yao, et al., Global emergence of a hypervirulent carbapenem-resistant *Escherichia coli* ST410 clone, Nat Commun. 15 (2024) 494. doi: 10.1038/s41467-023-43854-3.
[18] D. Gamal, M. Fernández-Martínez, I. El-Defrawy, A.A. Ocampo-Sosa, L. Martínez-Martínez, First identification of NDM-5 associated with OXA-181 in Escherichia coli from Egypt, Emerg Microbes Infect. 5 (2016) e30. doi: 10.1038/emi.2016.24.
[19] R. Patiño-Navarrete, I. Rosinski-Chupin, N. Cabanel, L. Gauthier, J. Takissian, J.Y. Madec, et al., Stepwise evolution and convergent recombination underlie the global dissemination of carbapenemase-producing *Escherichia coli*, Genome Med. 12 (2020) 10. doi: 10.1186/s13073-019-0699-6.
[20] A. Souvorov, R. Agarwala, D.J. Lipman, SKESA: strategic k-mer extension for scrupulous assemblies, Genome Biol. 19 (2018) 153. doi: 10.1186/s13059-018-1540-z.
[21] J. Hawkey, M.J. Loftus, A. Prasad, T. Vakatawa, V. Prasad, L. Tudravu, et al., Genomic diversity of clinically relevant bacterial pathogens from an acute care hospital in Suva, Fiji, JAC Antimicrob Resist. 7 (2025) dlaf058. doi: 10.1093/jacamr/dlaf058.
[22] S. Mahazu, I. Prah, A. Ayibieke, W. Sato, T. Hayashi, T. Suzuki, et al., Possible Dissemination of *Escherichia coli* Sequence Type 410 Closely Related to B4/H24RxC in Ghana, Front Microbiol. 12 (2021) 770130. doi: 10.3389/fmicb.2021.770130.
[23] N.R. Waters, F. Abram, F. Brennan, A. Holmes, L. Pritchard, Easy phylotyping of Escherichia coli via the EzClermont web app and command-line tool, Access Microbiol. 2 (2020) acmi000143. doi: 10.1099/acmi.0.000143.
[24] K.G. Joensen, A.M. Tetzschner, A. Iguchi, F.M. Aarestrup, F. Scheutz, Rapid and Easy *In Silico* Serotyping of *Escherichia coli* Isolates by Use of Whole-Genome Sequencing Data, J Clin Microbiol. 53 (2015) 2410-26. doi: 10.1128/jcm.00008-15.
[25] A. Carattoli, E. Zankari, A. Garcia-Fernandez, M. Voldby Larsen, O. Lund, L. Villa, et al., *In silico* detection and typing of plasmids using PlasmidFinder and plasmid multilocus sequence typing, Antimicrob Agents Chemother. 58 (2014) 3895-903. doi: 10.1128/aac.02412-14.
[26] J. Moussa, E. Nassour, T. Jisr, M. El Chaar, S. Tokajian, Characterization of bla(NDM-19)-producing IncX3 plasmid isolated from carbapenem-resistant *Escherichia coli* and *Klebsiella pneumoniae*, Heliyon. 10 (2024) e29642. doi: 10.1016/j.heliyon.2024.e29642.
[27] J.R. Grant, E. Enns, E. Marinier, A. Mandal, E.K. Herman, C.Y. Chen, et al., Proksee: in-depth characterization and visualization of bacterial genomes, Nucleic Acids Res. 51 (2023) W484-w92. doi: 10.1093/nar/gkad326.
[28] T.J. Carver, K.M. Rutherford, M. Berriman, M.A. Rajandream, B.G. Barrell, J. Parkhill, ACT: the Artemis Comparison Tool, Bioinformatics. 21 (2005) 3422-3. doi: 10.1093/bioinformatics/bti553.

Supplementary Figure S1.

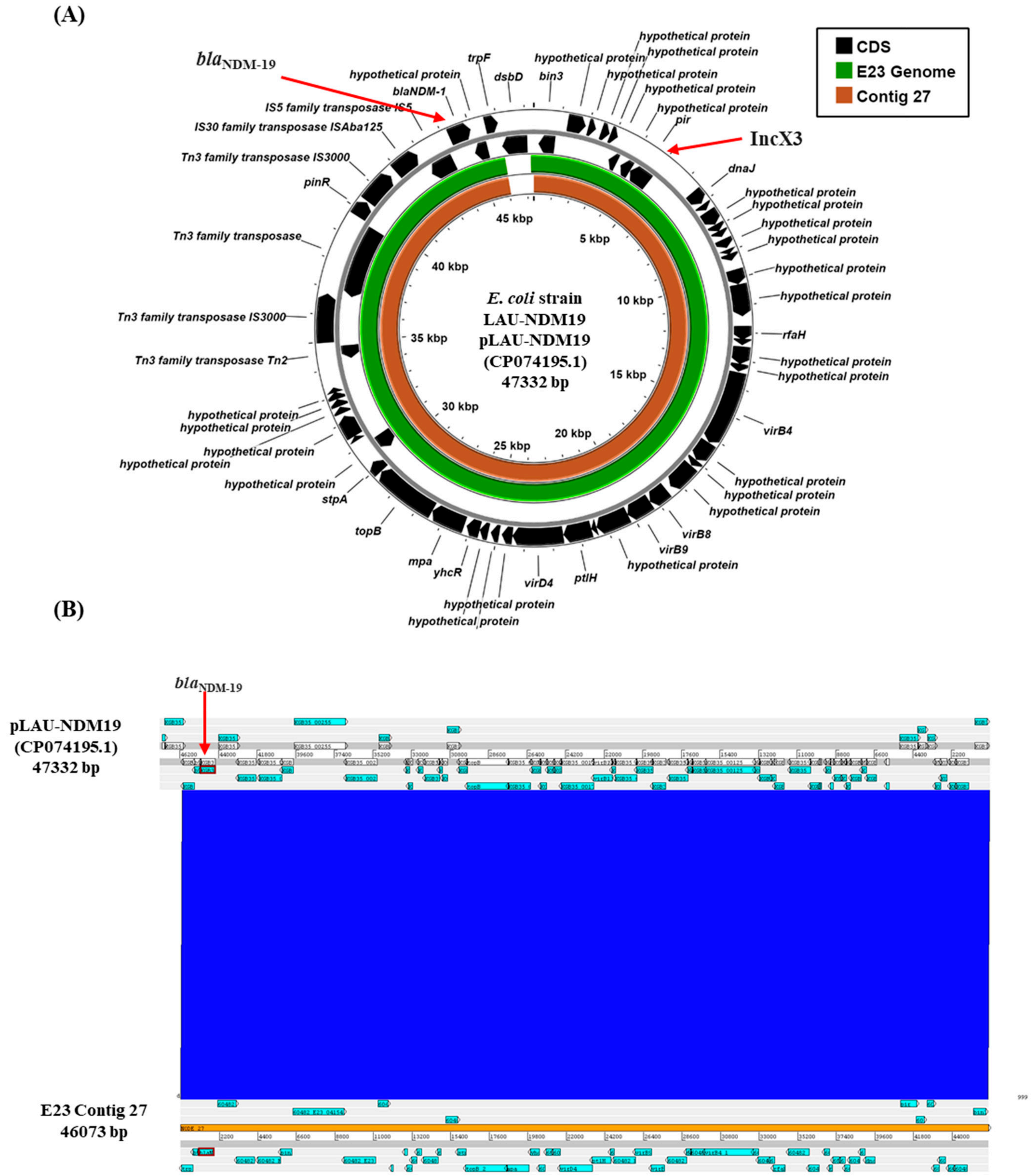

Supplementary Figure S1 (continued).

(C)

```
NDM-19_E23      MELPNIMHPVAKLSTALAAALMLSGCMPGEIRPTIGQQMETGDQRFGDLVFRQLAPNVWQ
NDM-19          MELPNIMHPVAKLSTALAAALMLSGCMPGEIRPTIGQQMETGDQRFGDLVFRQLAPNVWQ
NDM-7           MELPNIMHPVAKLSTALAAALMLSGCMPGEIRPTIGQQMETGDQRFGDLVFRQLAPNVWQ
NDM-1           MELPNIMHPVAKLSTALAAALMLSGCMPGEIRPTIGQQMETGDQRFGDLVFRQLAPNVWQ
NDM-5           MELPNIMHPVAKLSTALAAALMLSGCMPGEIRPTIGQQMETGDQRFGDLVFRQLAPNVWQ
                ************************************************************

NDM-19_E23      HTSYLDMPGFGAVASNGLIVRDGGRVLVVDTAWTDDQTAQILNWIKQEINLPVALAVVTH
NDM-19          HTSYLDMPGFGAVASNGLIVRDGGRVLVVDTAWTDDQTAQILNWIKQEINLPVALAVVTH
NDM-7           HTSYLDMPGFGAVASNGLIVRDGGRVLVVDTAWTDDQTAQILNWIKQEINLPVALAVVTH
NDM-1           HTSYLDMPGFGAVASNGLIVRDGGRVLVVDTAWTDDQTAQILNWIKQEINLPVALAVVTH
NDM-5           HTSYLDMPGFGAVASNGLIVRDGGRVLLVDTAWTDDQTAQILNWIKQEINLPVALAVVTH
                ***************************:********************************

NDM-19_E23      AHQDKMGGMNALHAAGIATYANALSNQLAPQEGLVAAQHSLTFAANGWVEPATAPNFGPL
NDM-19          AHQDKMGGMNALHAAGIATYANALSNQLAPQEGLVAAQHSLTFAANGWVEPATAPNFGPL
NDM-7           AHQDKMGGMNALHAAGIATYANALSNQLAPQEGLVAAQHSLTFAANGWVEPATAPNFGPL
NDM-1           AHQDKMGGMDALHAAGIATYANALSNQLAPQEGMVAAQHSLTFAANGWVEPATAPNFGPL
NDM-5           AHQDKMGGMDALHAAGIATYANALSNQLAPQEGLVAAQHSLTFAANGWVEPATAPNFGPL
                *********:***********************:**************************

NDM-19_E23      KVFYPGPGHTSDNITVGIDGTDIAFGGCLIKDSKAKSLGNLGDADTEHYAASVRAFGAAF
NDM-19          KVFYPGPGHTSDNITVGIDGTDIAFGGCLIKDSKAKSLGNLGDADTEHYAASVRAFGAAF
NDM-7           KVFYPGPGHTSDNITVGIDGTDIAFGGCLIKDSKAKSLGNLGDADTEHYAASARAFGAAF
NDM-1           KVFYPGPGHTSDNITVGIDGTDIAFGGCLIKDSKAKSLGNLGDADTEHYAASARAFGAAF
NDM-5           KVFYPGPGHTSDNITVGIDGTDIAFGGCLIKDSKAKSLGNLGDADTEHYAASARAFGAAF
                ****************************************************.*******

NDM-19_E23      PKASMIVMSHSAPDSRAAITHTARMADKLR
NDM-19          PKASMIVMSHSAPDSRAAITHTARMADKLR
NDM-7           PKASMIVMSHSAPDSRAAITHTARMADKLR
NDM-1           PKASMIVMSHSAPDSRAAITHTARMADKLR
NDM-5           PKASMIVMSHSAPDSRAAITHTARMADKLR
                ******************************
```

Supplementary Figure S2.

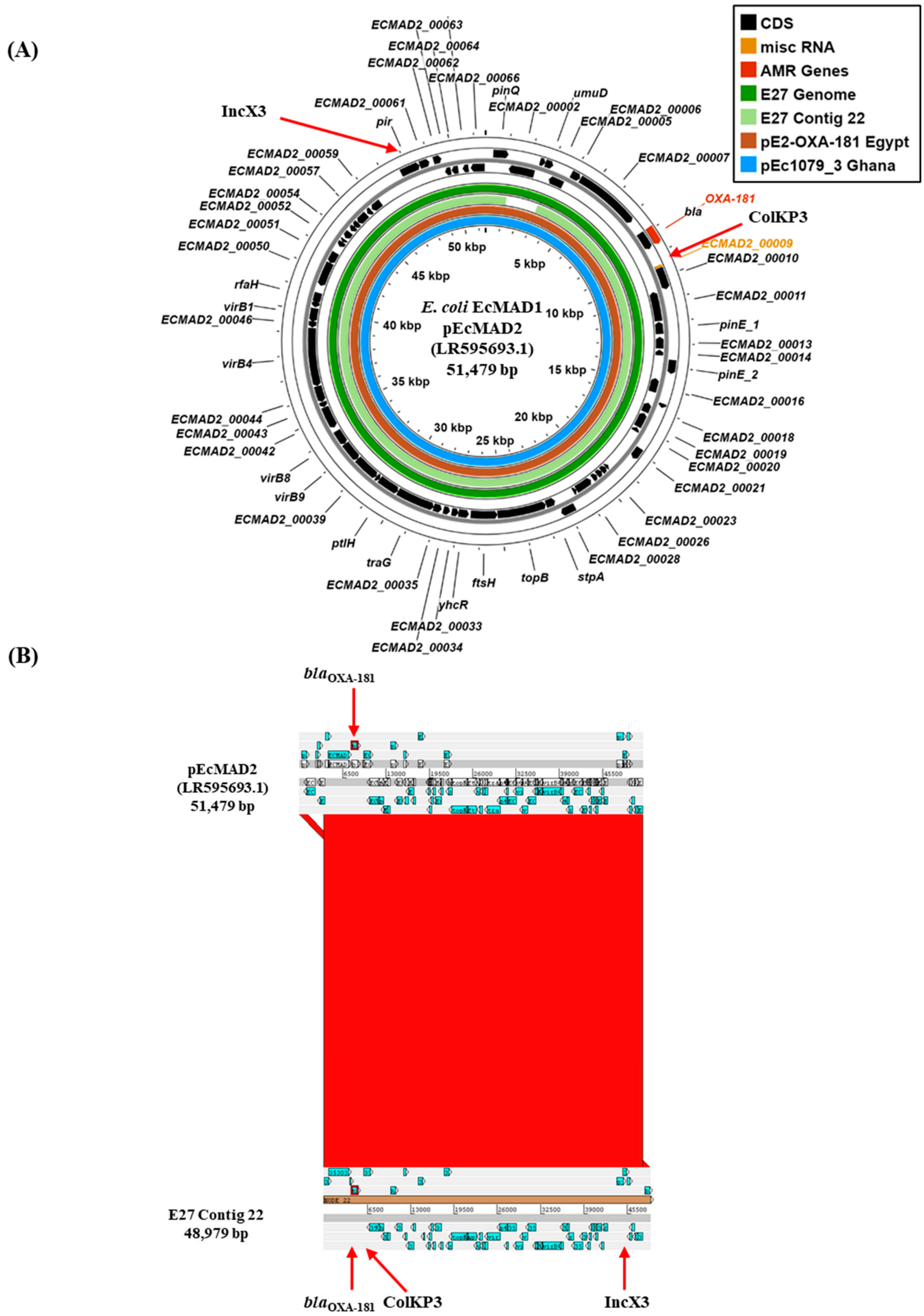

Supplementary Figure S3.

(A)

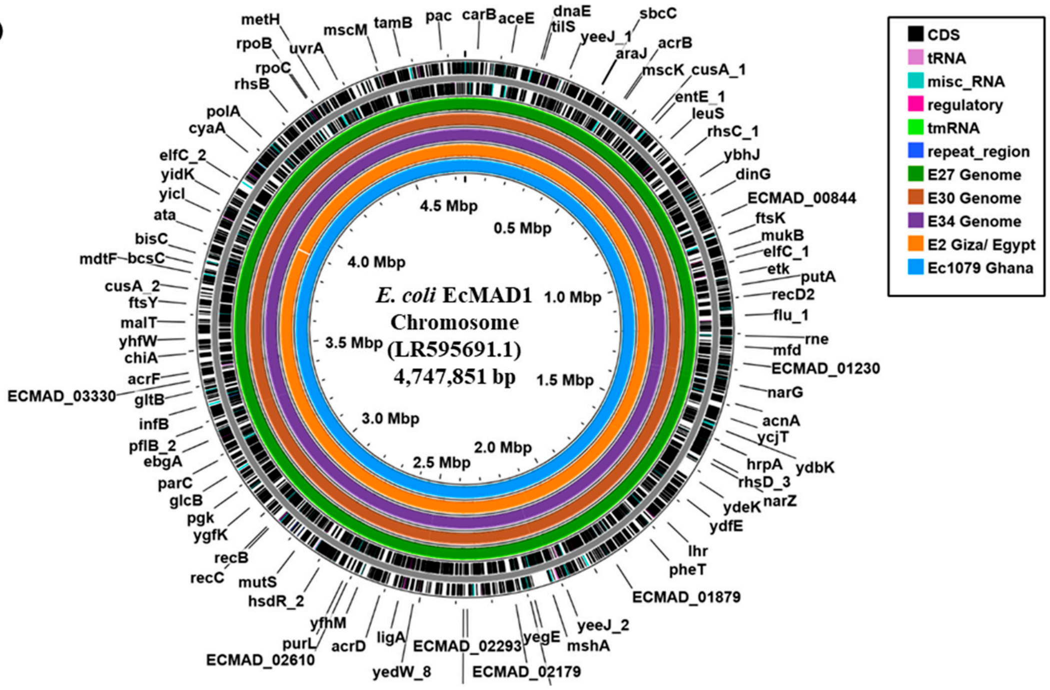

(B)

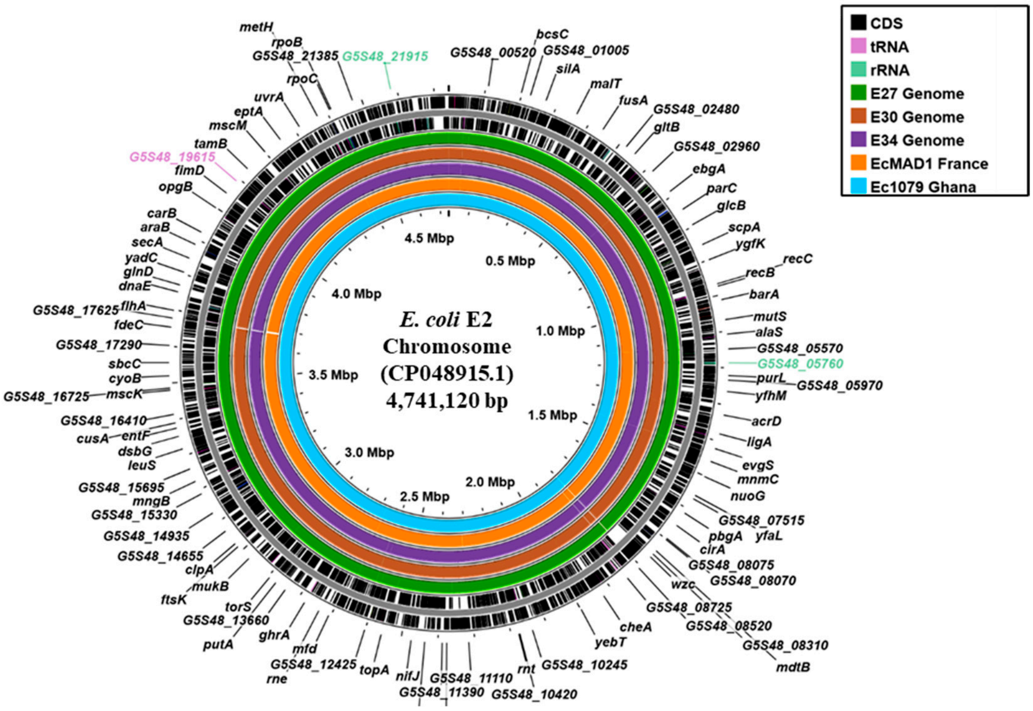

Supplementary Figure S4.

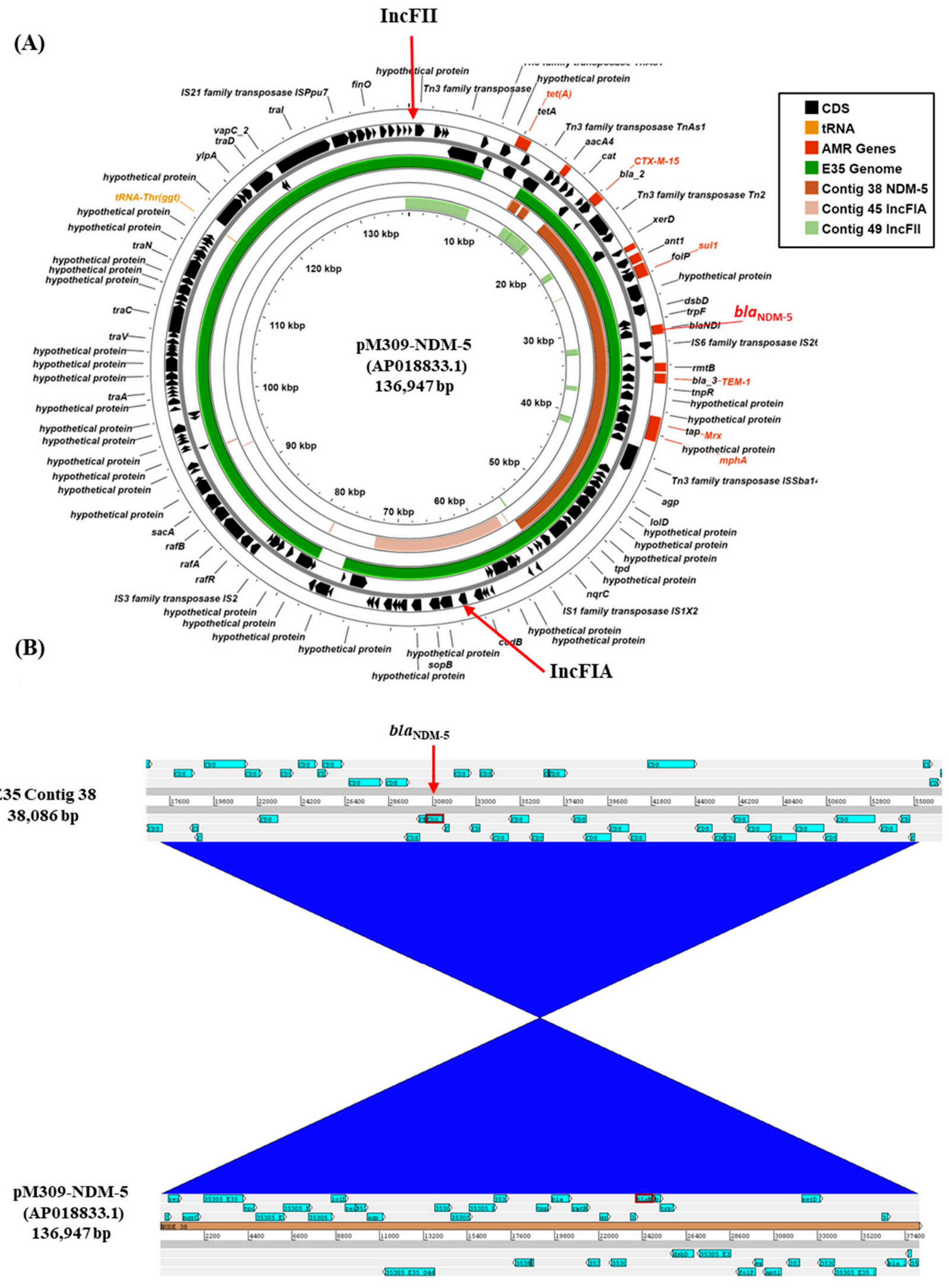

Supplementary Figure S5.

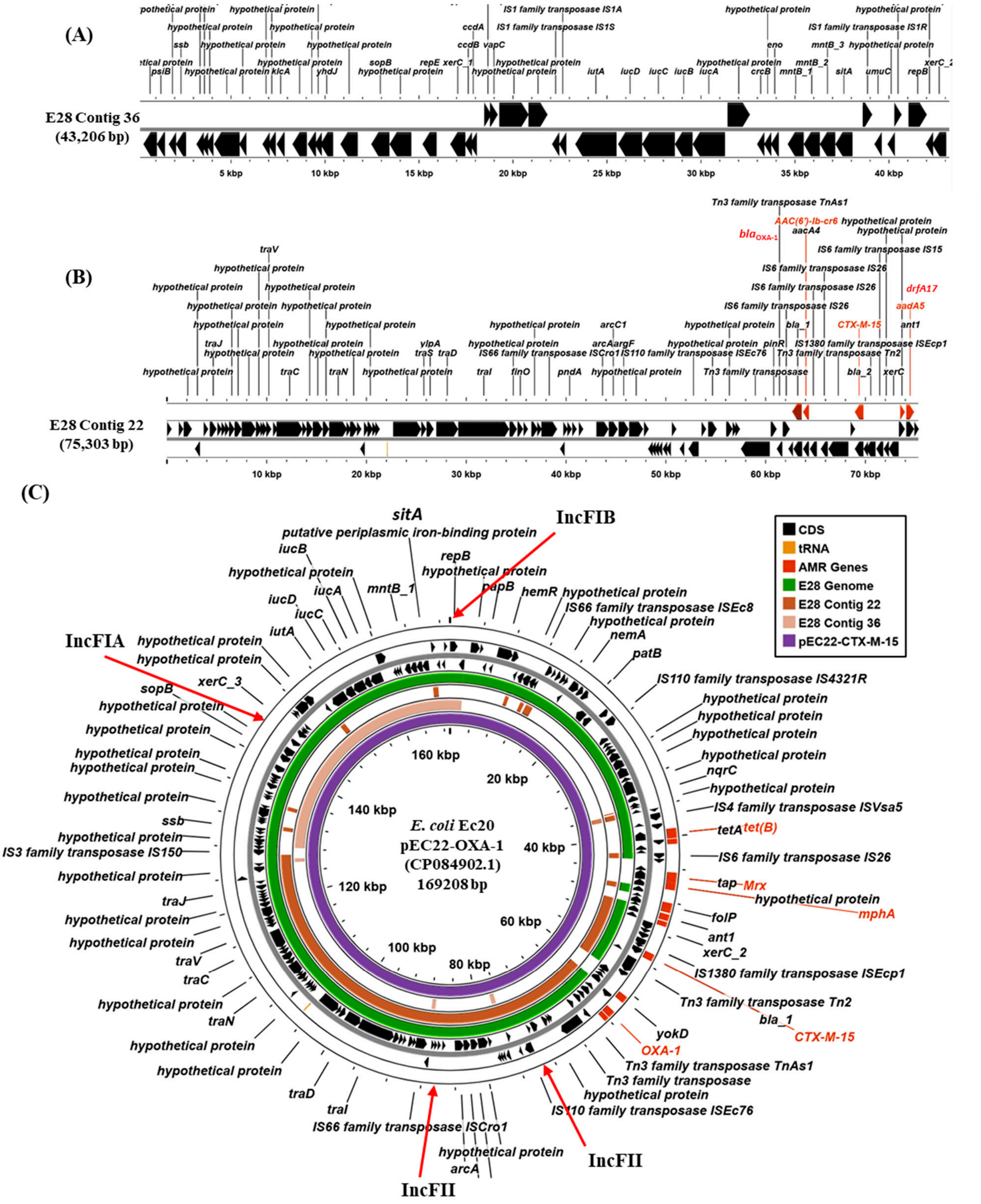

Supplementary Figure S5. (cont)

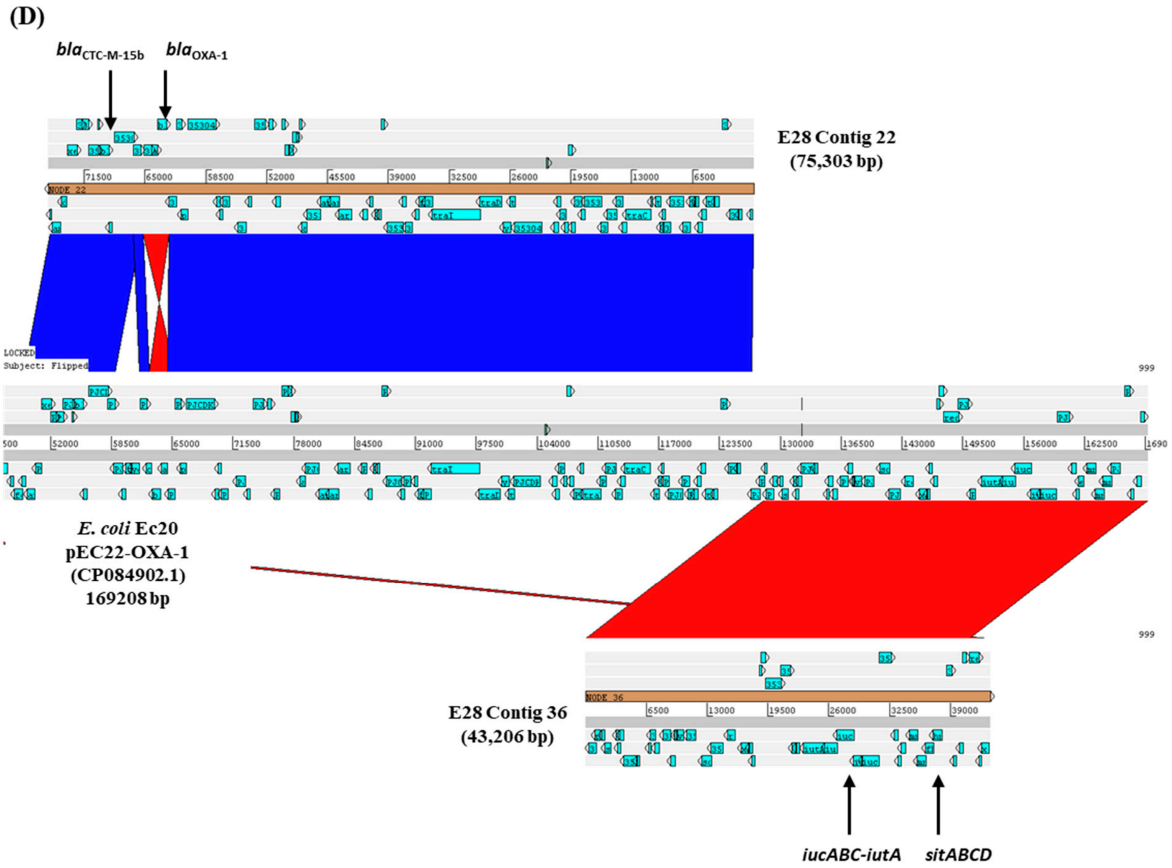

Supplementary Figure S6.

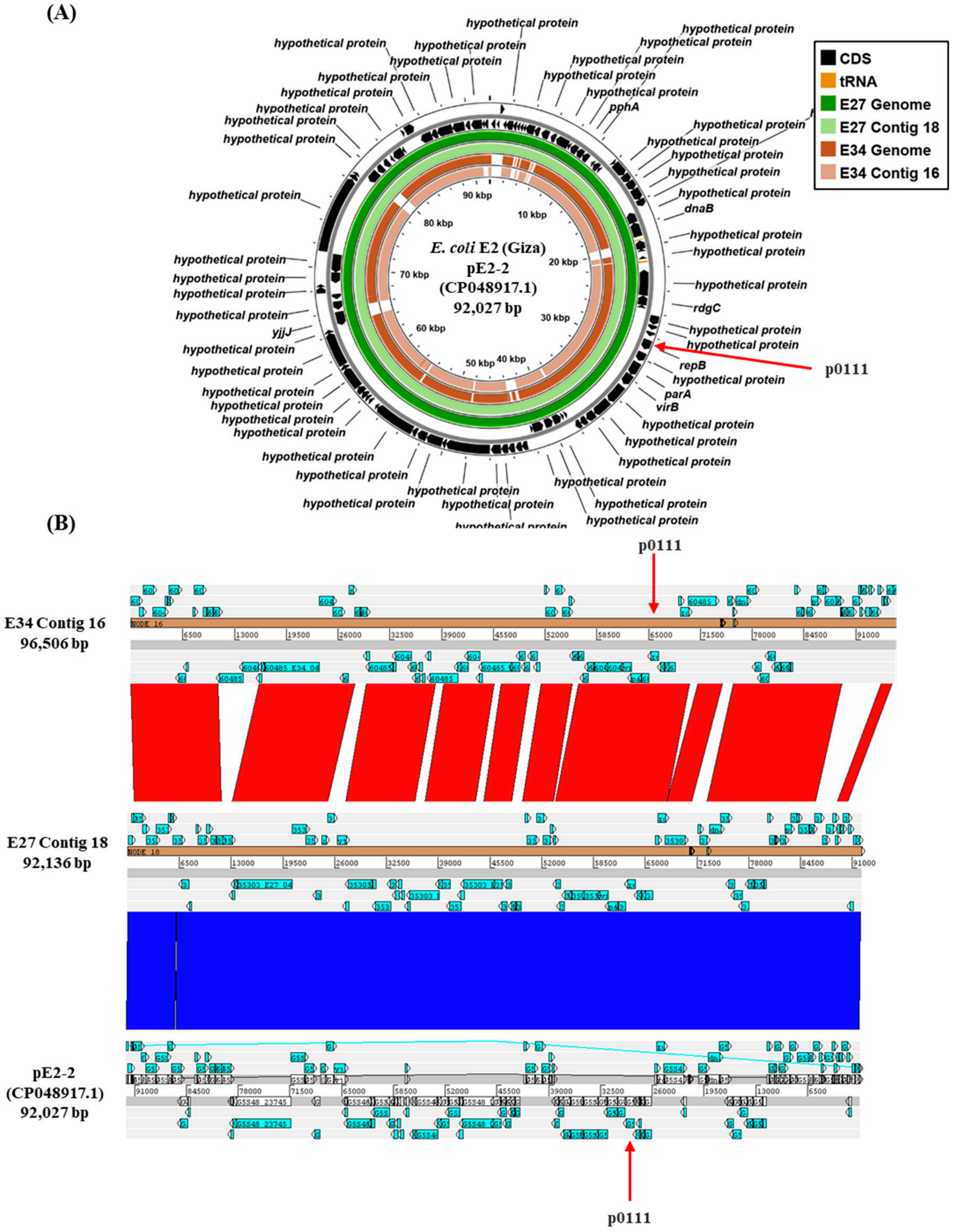

Supplementary Figure S7.

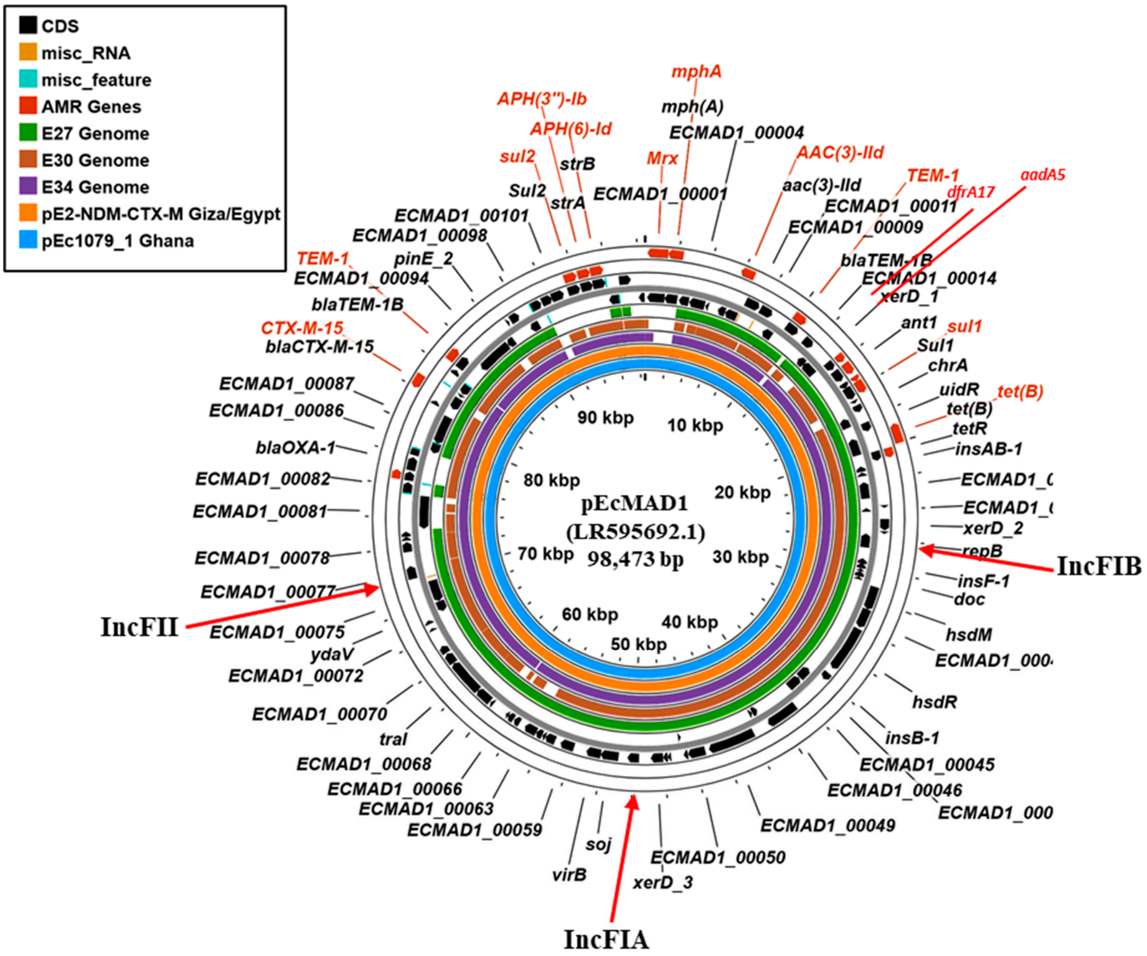

Supplementary Figure S8.

(A)

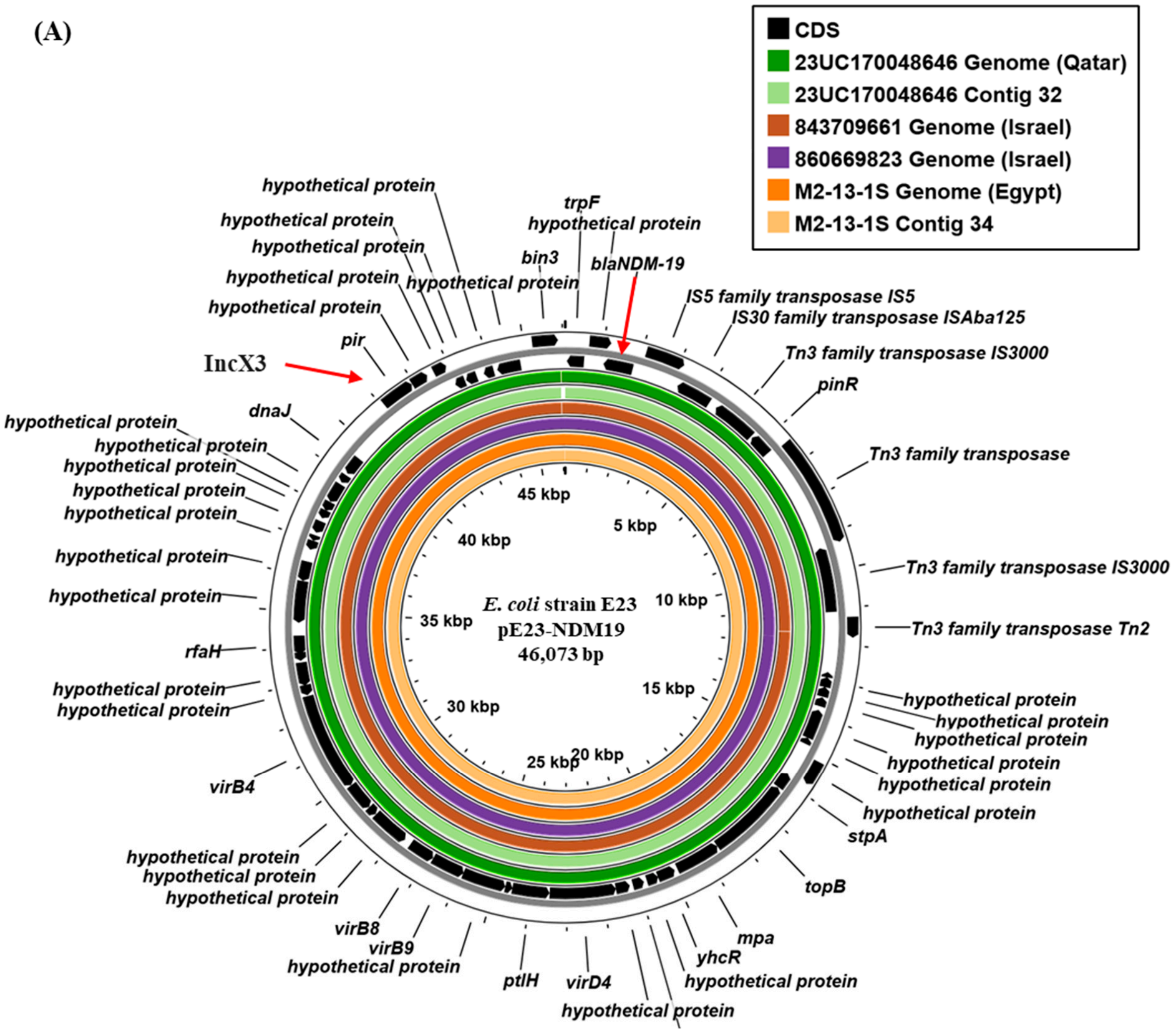

Supplementary Figure S8 (cont).

(B)

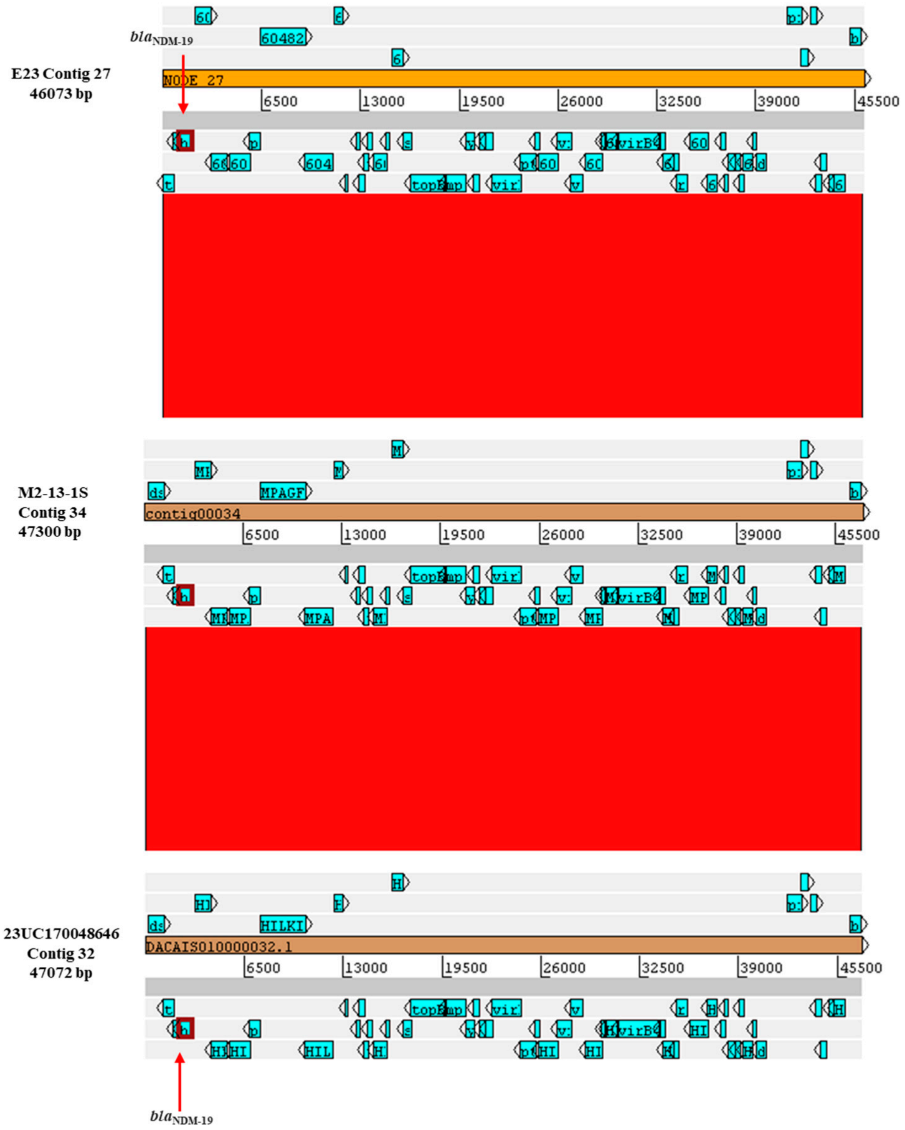

Supplement: Supplementary file 1 [file microorganisms-14-00247-s001.zip › microorganisms-4042951-supplementary.pdf]
